# Glomerular function in relation to fine airborne particulate matter in a representative population sample

**DOI:** 10.1038/s41598-021-94136-1

**Published:** 2021-07-19

**Authors:** Ying-Mei Feng, Lutgarde Thijs, Zhen-Yu Zhang, Esmée M. Bijnens, Wen-Yi Yang, Fang-Fei Wei, Bram G. Janssen, Tim S. Nawrot, Jan A. Staessen

**Affiliations:** 1 Department of Science and Technology, Beijing Youan Hospital, Capital Medical University, Beijing, 100069 China; 2 Research Unit Hypertension and Cardiovascular Epidemiology, KU Leuven Department of Cardiovascular Sciences, University of Leuven, Leuven, Belgium; 3 Center for Environment Sciences, Hasselt University, Diepenbeek, Belgium; 4 Shanghai General Hospital, Shanghai, China; 5 Department of Cardiology, The First Affiliated Hospital of Sun Yat-Sen University, Guangzhou, Guangdong China; 6 NHC Key Laboratory of Assisted Circulation, Sun Yat-Sen University, Guangzhou, China; 7 Non-Profit Research Institute Alliance for the Promotion of Preventive Medicine, Mechlin, Belgium; 8 Biomedical Sciences Group, Faculty of Medicine, University of Leuven, Leuven, Belgium

**Keywords:** Nephrology, Kidney, Kidney diseases

## Abstract

From 1990 until 2017, global air-pollution related mortality increased by 40%. Few studies addressed the renal responses to ultrafine particulate [≤ 2.5 µm (PM2.5)], including black carbon (BC), which penetrate into the blood stream. In a Flemish population study, glomerular filtration estimated from serum creatinine (eGFR) and the urinary albumin-to-creatinine ratio were measured in 2005–2009 in 820 participants (women, 50.7%; age, 51.1 years) with follow-up of 523 after 4.7 years (median). Serum creatinine, eGFR, chronic kidney disease (eGFR < 60 mL/min/1.73 m^2^) and microalbuminuria (> 3.5/> 2.5 mg per mmol creatinine in women/men) were correlated in individual participants via their residential address with PM_2.5_ [median 13.1 (range 0.3–2.9) μg/m^3^] and BC [1.1 (0.3–18) μg/m^3^], using mixed models accounting for address clusters. Cross-sectional and longitudinally, no renal outcome was associated with PM_2.5_ or BC in models adjusted for sex and baseline or time varying covariables, including age, blood pressure, heart rate, body mass index, plasma glucose, the total-to-HDL serum cholesterol ratio, alcohol intake, smoking, physical activity, socioeconomic class, and antihypertensive treatment. The subject-level geocorrelations of eGFR change with to BC and PM2.5 were 0.13 and 0.02, respectively (*P* ≥ 0.68). In conclusion, in a population with moderate exposure, renal function was unrelated to ultrafine particulate.

## Introduction

According to the report of the Global Burden of Disease Study, renal failure contributed to 0.403 million death in 1990 which increased to 0.736 million in 2010 worldwide^1^. The subsequent 2017 report confirmed this trend and highlighted the contribution of environmental pollutants, including air pollution, as driver of chronic kidney disease (CKD)^2^. From 1990 until 2017, global mortality and disability adjusted life-years (DALYs) related to ambient air pollution by particulate matter increased by 39.35% and 43.45%, respectively^2^.

Airborne fine particulate (PM_2.5_) reaches the smallest airways and alveoli, crosses the blood-air barrier and directly penetrates into the blood stream^3^. Black carbon (BC), a component of PM_2.5_, consists of pure carbon in several bond forms, and finds its origin in the incomplete combustion of fossil fuels, biofuel or biomass. The kidneys clear BC particles from the circulating blood^4^. The number of carbon particles in urine is measurable and reflects chronic exposure to combustion-related air pollution^4^. Several studies reported on the levels of particulate matters in relation to the glomerular filtration rate estimated from serum creatinine (eGFR)^5–8^, glomerulopathy^9^, prevalent^7^ or incident^10^ CKD, progression to end-stage renal disease^10^, microalbuminuria^11^, and the risk of renal cancer^12^ in relation to air pollutants. These studies enrolled population samples^11,12^, stroke patients^5^, or aged male veterans^6,8,10^. Some studies did not report the level of exposure^11^, or estimated exposure from the proximity of major roads^5^, while other findings were representative for exposure levels far above current air quality standards. Other reports focused on coarse particulate^7^. Of the eight reviewed studies^5–9,11,12^, none reported on BC exposure. To address this knowledge gap, we analysed data from The Flemish Study on Environment, Genes and Health Outcomes (FLEMENGHO), a family-based population study with catchment area in northern Belgium.

## Results

### Baseline characteristics of participants

All 820 participants were White Europeans, of whom 416 (50.7%) were women. Age at baseline (2005–2009) averaged (SD) 51.1 (15.6) years (5th–95th percentile interval, 23.6–75.8 years). Among 820 participants, 341 (41.6%) had hypertension, of whom 211 (61.9%) were on antihypertensive drug treatment, 75 (5.1%) had a history of cardiovascular disease, and 32 (3.9%) had diabetes. Among 211 patients on antihypertensive drug treatment, 18 (8.5%) took diuretics, 94 (44.6%) inhibitors of the renin system, 17 (8.1%) vasodilators, 2 (0.9%) centrally acting α_2_ adrenergic agonists and 80 (37.9%) were on combination therapy with more than one class of blood pressure lowering medication. Intake of statins was reported by 106 participants (12.9%). Serum creatinine and eGFR averaged 86.4 (15.5) µmol/L and 80.9 (16.4) mL/min/1.73m^2^, respectively and were approximately normally distributed (Supplementary Figure S1). The association between serum creatinine and age was curvilinear (Supplementary Figure S2). At baseline, 264 people (32.2%) were in CKD stage 1, 487 in CKD stage 2 (59.4%), 55 in CKD stage 3A (6.7%), 12 in CKD stage 3B (1.5%), 2 in CKD stage 4 (0.2%) and none in CKD stage 5. At baseline, the prevalence of microalbuminuria based on the urinary albumin-to-creatinine ratio was 14 (3.4%) in women and 15 (3.7%) in men.

Tables 1 and 2 list the characteristics of the participants by thirds of the distributions of BC and PM_2.5_, respectively. Most clinical and biochemical characteristics were similar across thirds of the BC and PM_2.5_ distributions, with the exception of plasma glucose which decreased from the lowest to the highest exposure group for both BC (*P* = 0.027) and PM_2.5_ (*P* = 0.033), and the prevalence of smoking (*P* = 0.027) which was higher in the top PM_2.5_ exposure group.

**Table 1:** Baseline characteristics of participants by thirds of the black carbon distribution.

Characteristic	0.74–1.07 μg/m^3^	1.04–1.30 μg/m^3^	1.15–1.95 μg/m^3^	*P*
**All in category**	273	273	274	
Women (n [%])	139 (50.9)	138 (50.6)	139 (50.7)	> 0.99
Smokers (n [%])	45 (16.5)	55 (20.2)	68 (24.8)	0.05
Alcohol intake ≥ 5 g/day (n [%])				
Hypertension (n [%])	112 (41.0)	113 (41.4)	116 (42.3)	0.95
Antihypertensive treatment (n [%])	70 (25.6)	67 (24.5)	74 (27.0)	0.80
Statin treatment (n [%])	40 (14.6)	32 (11.7)	34 (12.4)	0.56
Diabetes mellitus (n [%])	17 (6.2)	7 (2.6)	8 (2.9)	0.07
Cardiovascular disease (n [%])	12 (4.4)	9 (3.2)	18 (6.6)	0.19
Microalbuminuria (n [%])	6 (2.3)	15 (5.8)	8 (3.1)	0.09
CKD, stage ≥ 3 (n [%])	26 (9.5)	23 (8.4)	20 (7.3)	0.64
**Mean of characteristic**				
Age (years)	51.4 (15.4)	51.3 (15.6)	50.8 (16.0)	0.90
Body mass index (kg/m^2^)	26.7 (4.7)	26.3 (3.9)	26.6 (4.4)	0.63
Systolic pressure (mm Hg)	128.5 (17.6)	129.7 (16.9)	129.9 (17.9)	0.58
Diastolic pressure (mm Hg)	79.4 (9.9)	79.8 (9.0)	79.8 (9.7)	0.80
Mean arterial pressure (mm Hg)	95.7 (11.0)	96.5 (10.2)	96.5 (10.7)	0.63
Heart rate (beats per minute)	63.7 (9.9)	63.0 (9.4)	63.8 (9.9)	0.55
Biochemical data				
Serum creatinine (μmol/L)	86.2 (13.5)	86.4 (18.4)	86.8 (14.3)	0.91
eGFR (mL/min/1.73 m^2^)	80.6 (16.1)	80.9 (15.9)	81.3 (17.2)	0.88
Total cholesterol (mmol/L)	5.18 (0.92)	5.37 (1.04)*	5.21 (0.94)	0.06
HDL cholesterol (mmol/L)	1.40 (0.33)	1.45 (0.37)	1.43 (0.35)	0.22
Total-to-HDL cholesterol ratio	3.89 (1.02)	3.90 (1.09)	3.81 (0.99)	0.50
Plasma glucose (mmol/L)	5.05 (1.16)	4.91 (0.55)*	4.87 (0.56)	0.03
γ-glutamyltransferase (U/L)	22 (10–63)	2 (11–95)	22 (10–64)	0.89
Airborne particulate				
Black carbon (μg/m^3^)	0.99 (0.05)	1.12 (0.06)^§^	1.39 (0.22)^§^	< 0.0001
PM_2.5_ (μg/m^3^)	12.7 (0.27)	13.2 (0.34)^§^	14.5 (0.74)^§^	< 0.0001

CKD, chronic kidney disease; eGFR, glomerular filtration rate estimated from serum creatinine by the CKD-EPI formula; HDL, high-density lipoprotein. Microalbuminuria (> 3.5/> 2.5 mg per mmol creatinine in women/men) was available in 780 participants. Thirds of the black carbon distribution were determined after stratification for sex and age (< 50, 50–64, ≥ 65 years). Blood pressure was the average of five consecutive auscultatory readings. Hypertension was a blood pressure of ≥ 140 mm Hg systolic or ≥ 90 mm Hg diastolic, or use of antihypertensive drugs. Diabetes mellitus was a fasting plasma glucose of > 7.0 mmol/L (> 126 mg/dL) or use of antidiabetic agents. The central tendency (data spread) is given as arithmetic mean (SD) or median (5th–5th percentile interval). The central tendency (data spread) is given as arithmetic mean (SD) or median (5th–5th percentile interval). P-values were derived by Fisher exact test, ANOVA or the Kruskal–Wallis test. Significance of the difference with the adjacent lower third: *P ≤ 0.05; ^§^*P* ≤ 0.0001.

**Table 2:** Baseline characteristics of participants by thirds of the PM_2.5_ distribution.

Characteristic	11.0–13.0 μg/m^3^	12.9–13.9 μg/m^3^	13.3–16.1 μg/m^3^	*P*
**All in category**	273	273	274	
Women (n [%])	139 (50.9)	137 (50.2)	140 (51.1)	0.98
Smokers (n [%])	48 (17.6)	49 (18.0)	71 (25.9)*	0.03
Hypertension (n [%])	112 (41.0)	118 (43.2)	111 (40.5)	0.79
Antihypertensive treatment (n [%])	72 (26.4)	66 (24.2)	73 (26.6)	0.78
Statin treatment (n [%])	38 (13.9)	37 (13.6)	31 (11.3)	0.63
Diabetes mellitus (n [%])	17 (6.2)	7 (2.6)	8 (2.9)	0.07
Cardiovascular disease (n [%])	12 (4.4)	12 (4.4)	15 (5.5)	0.79
Microalbuminuria (n [%])	7 (2.7)	13 (5.1)	9 (3.4)	0.34
CKD, stage ≥ 3 (n [%])	26 (9.5)	25 (9.2)	18 (6.6)	0.40
**Mean of characteristic**				
Age (years)	51.6 (15.4)	51.7 (15.5)	50.2 (16.1)	0.47
Body mass index (kg/m^2^)	26.4 (4.3)	26.6 (4.4)	26.6 (4.4)	0.91
Systolic pressure (mm Hg)	128.2 (18.2)	130.7 (17.3)	129.2 (16.9)	0.26
Diastolic pressure (mm Hg)	79.2 (9.7)	80.0 (9.4)	79.9 (9.5)	0.56
Mean arterial pressure (mm Hg)	95.5 (11.1)	96.9 (10.6)	96.3 (10.2)	0.33
Heart rate (beats per minute)	63.5 (9.8)	63.0 (9.1)	64.0 (10.3)	0.46
Biochemical data				
Serum creatinine (μmol/L)	86.3 (13.4)	86.8 (19.3)	86.2 (13.0)	0.88
eGFR (mL/min/1.73 m^2^)	80.3 (15.9)	80.5 (16.1)	81.9 (17.2)	0.44
Total cholesterol (mmol/L)	5.19 (0.94)	5.33 (1.01)	5.24 (0.96)	0.24
HDL cholesterol (mmol/L)	1.40 (0.33)	1.42 (0.38)	1.45 (0.34)	0.40
Total-to-HDL cholesterol ratio	3.86 (1.00)	3.95 (1.09)	3.78 (1.00)	0.17
Plasma glucose (mmol/L)	5.04 (1.11)	4.92 (0.63)	4.86 (0.58)	0.03
γ-glutamyltransferase (units/L)	22 (10–67)	21 (10–95)	22 (11–63)	0.56
Airborne particulate				
Black carbon (μg/m^3^)	0.99 (0.06)	1.12 (0.07)^§^	1.39 (0.15)^§^	< 0.0001
PM_2.5_ (μg/m^3^)	12.7 (0.25)	13.2 (0.20)^§^	14.6 (0.70)^§^	< 0.0001

CKD, chronic kidney disease; eGFR, glomerular filtration rate estimated from serum creatinine by the CKD-EPI formula; HDL, high-density lipoprotein. Microalbuminuria (> 3.5/> 2.5 mg per mmol creatinine in women/men) was available in 780 participants. Thirds of the PM_2.5_ distribution were determined after stratification for sex and age (< 50, 50–64, ≥ 65 years). Blood pressure was the average of five consecutive auscultatory readings. Hypertension was a blood pressure of ≥ 140 mm Hg systolic or ≥ 90 mm Hg diastolic, or use of antihypertensive drugs. Diabetes mellitus was a fasting plasma glucose of > 7.0 mmol/L (> 126 mg/dL) or the use of antidiabetic agents. The central tendency (data spread) is given as arithmetic mean (SD) or median (5th–5th percentile interval). *P*-values were derived by Fisher exact test, ANOVA or the Kruskal–Wallis test. Significance of the difference with the adjacent lower third: *P ≤ 0.05; ^§^*P* ≤ 0.0001.

### Ambient air pollution

The median interval between the assessment of renal function and the midpoint assessment of long-term air pollution (30 June 2012) was 5.6 years (5th–95th percentile interval, 3.1–6.9 years) at baseline (2005–2009) and 0.4 years (0.6–2.6 years) at follow-up (2009–2013). Of the 820 participants, 231 lived alone, while 165, 33 and 38 shared a home with two, three or more participants. The median long-term air pollution levels (5th–95th percentile interval), to which participants were exposed, amounted to 1.10 μg/m^3^ (0.93–1.55 μg/m^3^) for BC and 13.1 μg/m^3^ (12.4–15.3 μg/m^3^) for PM_2.5_. While accounting for clustering among study participants sharing a residential address, the levels of the two air pollutants were highly correlated (r = 0.95).

### Cross-sectional analyses

In multivariable-adjusted cross-sectional analyses of all participants, serum creatinine, eGFR and the prevalence of CKD and microalbuminuria were unrelated to BC and PM_2.5_, irrespective of whether only the baseline data were used, only the follow-up data, or the baseline and follow-up data (Table 3, *P* ≥ 0.49). All models accounted for clustering of data among participants living at the same address and were adjusted for sex, age (linear and squared term), mean arterial pressure, heart rate, body mass index, plasma glucose, total-to-HDL cholesterol ratio, γ-glutamyltransferase as biomarker of alcohol consumption, smoking, daily energy expenditure in physical activity, socioeconomic class, and antihypertensive treatment (by drug class). A correlation matrix between continuously distributed covariables measured at baseline was presented in Supplementary Table S1 and Supplementary Table S2 for men and women, respectively. In line with that, the characteristics of the participants by sex, age, smoking status and daily alcohol intake was presented in Supplementary Table S3, S4, S5 and S6, respectively.

**Table 3:** Multivariable-adjusted cross-sectional associations of renal function with exposure to black carbon and PM_2.5_.

Airborne particulate Renal function	Baseline only (n = 820)		Follow-up only (n = 653)		Baseline and follow-up (n = 820)	
	Estimate (95% CI)	*P*	Estimate (95% CI)	P	Estimate (95% CI)	*P*
**Black carbon**						
Serum creatinine (μmol/L)	0.35 (− 0.93 to 1.64)	0.59	0.29 (− 1.84 to 2.42)	0.79	0.39 (− 1.08 to 1.85)	0.61
eGFR (mL/min/1.73 m^2^)	− 0.08 (− 1.25 to 1.09)	0.90	− 0.37 (− 1.85 to 1.10)	0.62	− 0.04 (− 1.20 to 1.12)	0.95
Prevalence						
Chronic kidney disease (OR)	− 0.09 (− 0.43, 0.24)	0.59	0.12 (− 0.22 to 0.45)	0.49	0.03 (− 0.25 to 0.31)	0.85
Microalbuminuria (OR)	0.20 (− 0.35 to 0.75)	0.48	0.15 (− 0.40 to 0.71)	0.59	0.23 (− 0.22 to 0.67)	0.32
**PM**_**2.5**_						
Serum creatinine (μmol/L)	0.23 (− 1.07 to 1.52)	0.73	0.10 (− 2.03 to 2.23)	0.93	0.28 (− 1.19 , 1.75)	0.71
eGFR (mL/min/1.73 m^2^)	0.00 (− 1.18 to 1.19)	0.99	− 0.30 (− 1.78 to1.18)	0.69	0.01 (− 1.16 to 1.17)	0.99
Prevalence						
Chronic kidney disease (OR)	− 0.09 (− 0.42 to 0.24)	0.59	0.04 (− 0.29 to 0.38)	0.81	− 0.01 (− 0.29 to 0.26)	0.94
Microalbuminuria (OR)	0.27 (− 0.26 to 0.79)	0.32	0.06 (− 0.43 to 0.54)	0.82	0.21 (− 0.19 to 0.61)	0.31

eGFR is the glomerular filtration rate estimated from serum creatinine by the CKD-EPI formula. Associations sizes were derived from mixed models, which accounted for clustering of the baseline and follow-up data among participants living at the same address. For chronic kidney disease (eGFR < 60 mL/min/1.73 m^2^) and microalbuminuria (> 3.5/> 2.5 mg per mmol creatinine in women/men), association sizes are expressed as odds ratios (OR). Models were adjusted for sex, age (linear and squared term), mean arterial pressure, heart rate, body mass index, plasma glucose, total-to-HDL cholesterol ratio, γ-glutamyltransferase, smoking, daily energy expenditure in physical activity, socioeconomic class, and antihypertensive treatment (by drug class). Association sizes, given with 95% confidence interval, were expressed for an interquartile range increment in the airborne particulate.

### Renal function changes

**Figure 1:**
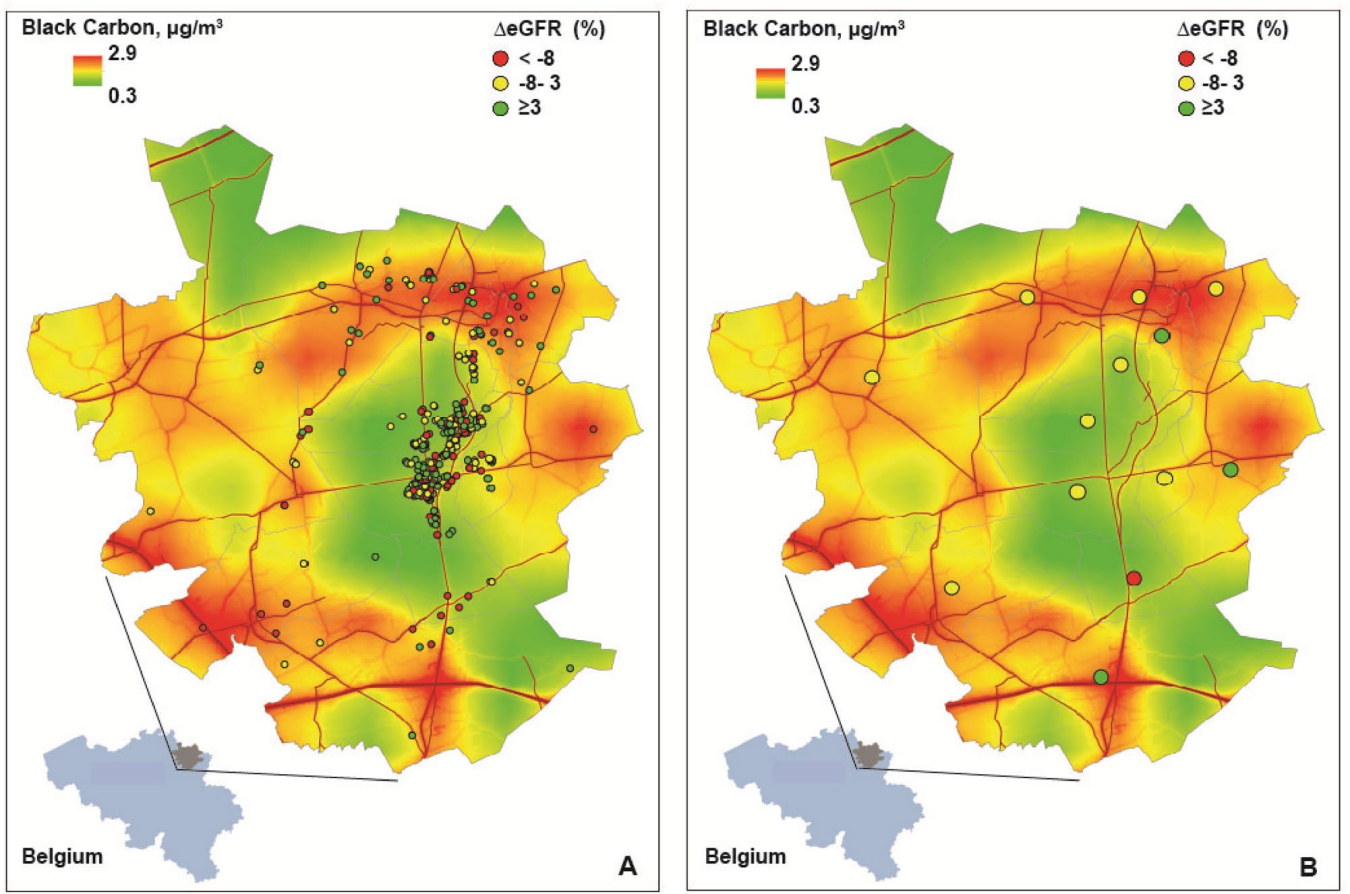
Geographical associations of the multivariable-adjusted percent change in the glomerular filtration rate estimated from serum creatinine (ΔeGFR) at the individual level (**A**) or aggregated per municipality (**B**) with black carbon air pollution contours. Grey lines indicate borders of municipalities. Red lines represent the air pollution contours of major roads. The Spearman rank correlation coefficients between percent change in eGFR and the exposure to BC were -0.016 (*P* = 0.68) and 0.200 (*P* = 0.52) in the individual and aggregated data, respectively.

**Figure 2:**
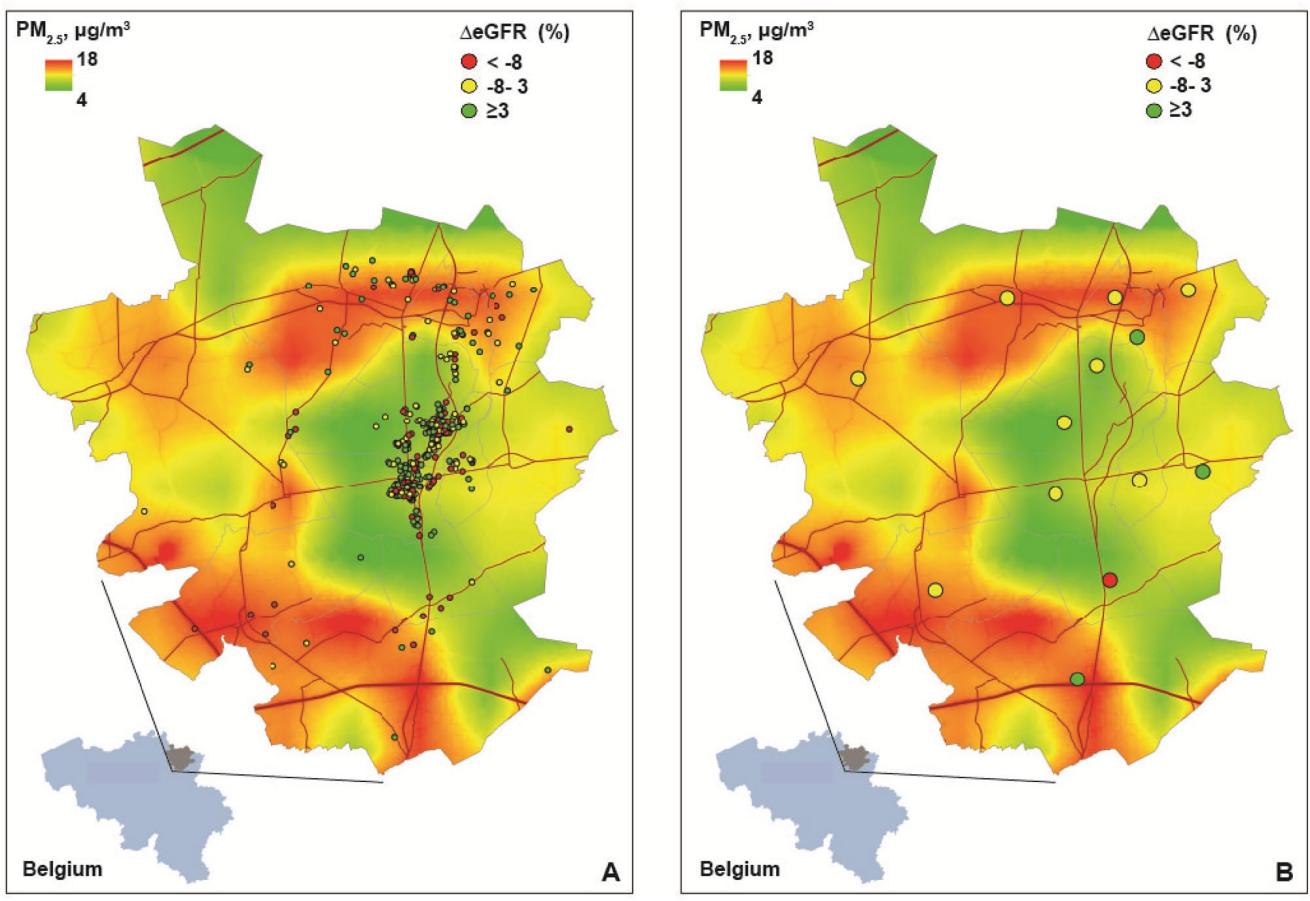
Geographical associations of the multivariable-adjusted percent change in the glomerular filtration rate estimated from serum creatinine (ΔeGFR) at the individual level (**A**) or aggregated per municipality (**B**) with PM_2.5_ air pollution contours. Grey lines indicate borders of municipalities. Red lines represent the air pollution contours of major roads. The Spearman rank correlation coefficients between percent change in eGFR and the exposure to PM_2.5_ were − 0.016 (*P* = 0.69) and − 0.022 (*P* = 0.94) in the individual and aggregated data, respectively.

Table 4 lists the baseline and follow-up characteristics, and the changes from baseline to follow, in the 653 participants who had a follow**-**up measurement of renal function. After a median of 4.8 years (5th to 95th percentile interval, 3.7–5.4 years) serum creatinine increased by 3.7 µmol/L (*P* < 0.0001) and eGFR decreased by 1.9 mL/min/1.73 m^2^ (*P* < 0.0001). From baseline to follow-up, eGFR reverted back to a value > 60 mL/min/1.73 m^2^ in 7 participants with CKD at baseline and microalbuminuria disappeared in 2. Of the 600 participants with CKD stage ≤ 2 at baseline, 48 progressed to CKD stage ≥ 3 at follow-up (*P* = 0.0004); among 549 participants without microalbuminuria at baseline, 9 developed microalbuminuria (*P* = 0.038). In longitudinal analyses (Table 5), we accounted for baseline serum creatinine and baseline eGFR by calculating the percent change between the repeat and the first measurement of these biomarkers, while adjusting the mixed models for covariables, including sex, baseline age (linear and square term), socioeconomic class, follow-up duration, baseline body mass index, and the baseline value of and change during follow-up in mean arterial pressure, heart rate, plasma glucose, the total-to-HDL serum cholesterol ratio, γ-glutamyltransferase, smoking status, and the intake of antihypertensive drugs (all drugs combined). The changes in serum creatinine, eGFR and the prevalence of CKD and microalbuminuria were unrelated to BC and PM_2.5_ (Table 5; *P* ≥ 0.48). The geographical associations of the multivariable-adjusted percent change in the eGFR in relation to BC and PM2.5 appear in Figs. 1 and 2, respectively.

**Table 4:** Baseline and follow-up characteristics of 653 participants.

Characteristic	Baseline 2005–2009	Follow-up 2009–2013	Change 95% CI
**Number with characteristic**			
Women (n [%])	328 (50.2)	328 (50.2)	–
Smokers (n [%])	124 (19.0)	98 (15.0)	− 4.0 (− 5.6, − 2.3)^§^
Alcohol intake ≥ 5 g/day (n [%])			
Hypertension (n [%])	268 (41.0)	335 (51.3)	10.3 (7.0, 13.5)^§^
Antihypertensive treatment (n [%])	163 (25.0)	212 (32.5)	7.5 (5.1, 9.9)^§^
Diabetes mellitus (n [%])	23 (3.5)	43 (6.6)	3.1 (1.7, 4.4)^§^
CKD, stage ≥ 3 (n [%])	53 (8.2)	94 (14.4)	6.2 (4.1, 8.5)^§^
Microalbuminuria (n [%])	17 (3.0)	24 (4.2)	1.2 (0.05, 2.6)*
**Mean of characteristic**			
Age (years)	50.9 (14.7)	55.6 (14.6)	4.7 (4.7, 4.8)^§^
Body mass index (kg/m^2^)	26.5 (4.4)	27.3 (4.4)	0.7 (0.6, 0.9)^§^
Systolic pressure	128.7 (16.7)	132.2 (16.8)	3.5 (2.4, 4.6)^§^
Diastolic pressure	79.8 (9.4)	82.3 (9.7)	2.5 (1.8, 3.2)^§^
Mean arterial pressure	96.1 (10.3)	98.9 (10.1)	2.8 (2.1, 3.5)^§^
Heart rate (beats per minute)	62.7 (9.4)	62.2 (9.7)	− 0.5 (− 1.3, 0.3)
Biochemical data			
Serum creatinine (μmol/L)	86.4 (15.5)	90.0 (22.7)	3.7 (2.6, 4.7)^§^
eGFR (mL/min/1.73 m^2^)	80.9 (15.4)	79.0 (18.1)	− 1.9 (− 2.8, − 1.0)^§^
Total cholesterol (mmol/L)	5.3 (0.96)	5.0 (0.95)	− 0.25 (− 0.32, − 0.18)^§^
HDL-cholesterol (mmol/L)	1.43 (0.35)	1.45 (0.38)	0.02 (0.01, 0.04)*
Total-to-HDL cholesterol ratio	3.85 (1.01)	3.68 (1.24)	− 0.15 (− 0.32, 0.02)
Plasma glucose (mmol/L)	4.94 (0.83)	4.96 (0.79)	− 0.00 (− 0.08, 0.07)
γ-glutamyltransferase (U/L)	23.3 (10–69)	24.7 (11–72)	3.7 (0.3, 7.2)*

CKD, chronic kidney disease; eGFR, estimated glomerular filtration rate derived from serum creatinine by the CKD-EPI formula; HDL, high-density lipoprotein. Blood pressure was the average of five consecutive auscultatory readings. Hypertension was a blood pressure of ≥ 140 mm Hg systolic or ≥ 90 mm Hg diastolic, or use of antihypertensive drugs. Diabetes mellitus was a fasting plasma glucose of > 7.0 mmol/L (> 126 mg/dL) or use of antidiabetic agents. The assessment of microalbuminuria (> 3.5/> 2.5 mg per mmol creatinine in women/men) was available in 566 participants. The central tendency (data spread) is given as arithmetic mean (SD) or median (5th-5th percentile interval). For categorical variables, percentage change is given. Significance of the change from baseline to follow-up (95% confidence interval): *P ≤ 0.05; ^†^*P* ≤ 0.01; ^‡^*P* < 0.001; ^§^*P* ≤ 0.0001.

**Table 5:** Multivariable-adjusted associations between changes from baseline to follow-up in renal function and in the exposure to airborne particulate.

Renal function trait	N	Black carbon		PM_2.5_	
		Estimate (95% CI)	*P*	Estimate (95% CI)	*P*
**Continuous outcomes**					
Serum creatinine (%)	653	0.10 (− 1.55 to 1.76)	0.90	0.25 (− 1.42, 1.92)	0.77
eGFR (%)	653	− 0.45 (− 1.92, 1.00)	0.54	− 0.53 (− 1.99, 0.93)	0.48
**Incidence**					
CKD (OR)	48/600	0.13 (− 0.25 to 0.50)	0.51	0.02 (− 0.36 to 0.39)	0.93
Microalbuminuria (OR)	9/549	0.09 (− 0.99 to 1.16)	0.88	0.06 (− 0.84 to 0.95)	0.90

eGFR is the glomerular filtration rate estimated from serum creatinine by the CKD-EPI formula. For continuously distributed variables (serum creatinine and eGFR), changes were computed as the follow-up minus baseline values and expressed as a percentage of the baseline value. Incidence refers to new-onset chronic kidney disease (< 60 mL/min/1.73 m^2^). N indicates the number of participants in the analysis or the number of new-onset cases/number of participants at risk. Associations accounted for clustering of data among participants living at the same address and were adjusted for sex, baseline age (linear and square term), socioeconomic class, follow-up duration, baseline body mass index, and the baseline value of and change during follow-up in mean arterial pressure, heart rate, plasma glucose, the total-to-HDL serum cholesterol ratio, γ-glutamyltransferase, smoking status, and the intake of antihypertensive drugs (all drugs combined). Association sizes were expressed for an interquartile range increment in the airborne particulate.

The Spearman rank correlation coefficients between percent change in eGFR and the exposure to BC were − 0.016 (*P* = 0.68) and + 0.20 (*P* = 0.52) in the individual data and in the data aggregated by municipality (Fig. 1). For the PM2.5 exposure, the corresponding estimates were − 0.016 (*P* = 0.69) and − 0.022 (*P* = 0.94), respectively (Fig. 2).

### Sensitivity analyses

The subgroup analyses, dichotomised by sex, age, smoking status and daily alcohol consumption produced consistent results. This was the case for the cross-sectional analyses (Supplementary Table S7 and Supplementary Table S8) and the longitudinal analyses (Supplementary Table S9 and Supplementary Table S10) in relation to BC (Supplementary Table S7 and Supplementary Table S9) and PM2.5 (Supplementary Table S8 and Supplementary Table S10). Along similar lines, the cross-section and longitudinal analyses with reduced adjustment were confirmatory (Supplementary Table S11 and Supplementary Table S12). The covariables kept in these analyses were sex, age (linear and squared term) and antihypertensive treatment (all drug classes combined) in the cross-sectional approach. In the longitudinal analyses the covariables were sex, baseline age (linear and square term), follow-up duration and the intake of antihypertensive drugs at baseline and follow-up (all drug classes combined).

## Discussion

In the current population study, we evaluated the associations of eGFR and change in eGFR with BC and PM_2.5_. The prospective analyses demonstrated over nearly 5 years a decline in eGFR of approximately 2 mL/min/1.73 m^2^ and a progression from CKD stage 2 or less to CKD stage 3 or higher in nearly 8% of participants. However, findings with regard to the key research question were negative. There was no association of eGFR, change in eGFR or CKD progression with BC or PM_2.5_. The current findings contrast with the observation that in the same study cohort (N = 671), systolic left ventricular function was slightly worse (*P* ≤ 0.027) with higher BC and PM2.5 exposure with association sizes per interquartile range increment ranging from − 0.339 to − 0.458% for longitudinal strain and from − 0.033 to − 0.049 s^−1^ for the longitudinal strain rate^13^^.^ In the Environmental Influence on Early Ageing study (ENVIRONAGE)^14^, in 381 mother–offspring pairs, an interquartile range increment in PM2.5 exposure over the entire pregnancy was positively associated with mitochondrial DNA methylation (*MT-RNR1*: + 0.91%, *P* = 0.01 and D-loop: + 0.21%, *P* = 0.05) and inversely associated with mitochondrial DNA content in placental tissue (relative change of − 15.6%, *P* = 0.001). Taken together, these divergent findings must be interpreted within the context of the circulatory kinetics of the ultrafine particulate. The PM_2.5_ fraction and the nano-sized BC particles readily penetrate the air-blood barrier in the lung alveoli and are subsequently dispersed body wide, as evidenced by the detection of BC particles in urine^4^. However, the heart is the first target organ on the circulatory path of these toxic particles. The uterine arteries are tributaries branching off directly from the internal iliac artery. At term, maternal blood flow to the placenta is within the 600–700 mL/min range and the length of the foetal capillaries is close to 320 kilometres^15^. The renal blood flow represents 20% of cardiac output, but over 90% of the renal blood flow is distributed to the cortex^16^. These haemodynamic properties, the downstream dilution of the toxic particles from their point of entry into the circulation, and the capillary surface exposed probably explain the divergent vulnerability of organs.

Particulate matter is a mixture of solid and liquid particles in the air^17^. Most previous studies focused on PM_10_^5–12^. In 3901 participants enrolled in the Multi-Ethnic Study of Atherosclerosis, the urinary albumin**-**to**-**creatinine ratio was measured at three visits 1.5 to 2 years apart from baseline (2000–2004) and correlated with PM_10_ as measured by ambient air pollution monitors over 1 month, 2 months and 20 years before the first visit, with adjustments applied for race/ethnicity, sex, age smoking, second-hand smoking, body mass index and dietary protein intake. The study did not demonstrate a significant association between prevalent microalbuminuria or accelerated development of microalbuminuria in relation to PM_10_^11^. In contrast, among 1103 consecutive Boston-area patients hospitalised with acute ischaemic stroke between 1999 and 2004, eGFR was correlated with residential distance to major roadways ranging from 50 m or less to over 1 km with adjustments applied for race, sex, age, smoking, comorbid conditions, treatment with angiotensin-converting enzyme inhibitors, and neighbourhood-level socioeconomic status^5^. Patients living closer to a major roadway had lower eGFR than patients living farther away (P for trend = 0.01). Patients living at a distance of 50 m *vs.* 1 km had a 3.9 mL/min/1.73 m^2^ lower eGFR (*P* = 0.007)^5^. Among 21,656 residents of Taipei (2007–2009)^7^, eGFR and the prevalence of CKD (eGFR < 60 mL/min/1.73 m^2^) were correlated with PM_coarse_, PM_10_ and PM_2.5_ with adjustments applied for sex, age, body mass index, hypertension (categorical), fasting blood glucose, total serum cholesterol, smoking and drinking, educational attainment, and distance to a major roads (continuous). eGFR was 0.69 mL/min/1.73 m^2^ lower per interquartile range increment in PM_10_ (5.83 μg/m^3^), while the odds of having CKD were 1.15 times higher for the same increment in PM_10_^7^. The corresponding estimates for PM_coarse_ (6.59 μg/m^3^) were − 1.07 mL/min/1.73 m^2^ for eGFR and 1.26 for CKD. In contrast to the associations with PM_10_ and coarse PM, those with PM_2.5_ did not reach statistical significance^7^.

Human studies relating renal function to exposure to PM_2.5_ reported conflicting results^6,7,9,10^. In the cross-sectional Taiwanese study reviewed above^7^, there was no association between eGFR or CKD with PM_2.5_ (annual average concentration, 26.6 μg/m^3^). In a longitudinal analysis of 669 men enrolled in the Veterans Administration Normative Aging Study with up to four visits between 2000 and 2011 (n = 1715 visits)^6^, 1-year exposure to PM_2.5_ prior to each visit was assessed using a validated spatiotemporal model that utilised satellite remote**-**sensing aerosol optical depth data. In all person**-**visits by calendar year, the mean yearly average PM_2.5_ concentrations increased from 10.5 μg/m^3^ in 2000 to their highest level of 11.8 μg/m^3^ in 2002 and later decreased to less than 9.0 μg/m^3^ in 2010 and 2011. eGFR was analysed by time-varying linear mixed-effects regression models as continuous function of the 1-year PM_2.5_ levels, while adjusting for a large number of covariables. A 2.1-μg/m^3^ (interquartile range) higher 1**-**year PM_2.5_ was associated with a 1.87 mL/min/1.73 m^2^ lower eGFR (95% confidence interval, 0.76–2.99 mL/min/1.73 m^2^). A 2.1 μg/m^3^ higher 1**-**year PM_2.5_ was also associated with an additional annual decrease in eGFR of 0.60 mL/min/1.73 m^2^ per year (95% confidence interval, 0.40–0.79 mL/min/1.73 m^2^). A recent study linked the Environmental Protection Agency and the Department of Veterans Affairs databases to build an observational cohort of 2,482,737 United States veterans^10^. It applied survival models to evaluate the association of PM_2.5_ concentrations and risk of incident CKD and end-stage renal disease^10^. County**-**level exposure was defined at baseline as the annual average PM_2.5_ concentrations in 2004, and as a time**-**varying variable updated annually and as cohort participants moved. In analyses of baseline exposure (median, 11.8 μg/m^3^ [interquartile range10.1–13.7 μg/m^3^]), a 10-μg/m^3^ increase in PM_2.5_ concentration was associated with a 20% to 30% increase in the risk of CKD and end-stage renal dysfunction. The time**-**varying analyses produced similar results^10^. A Chinese study of 71,151 renal biopsies involving 282 cities with standardisation for age and region identified IgA nephropathy (28.1%) and membranous nephropathy (23.1%) as the leading causes of nephropathy^9^. Three-year average PM_2.5_ exposure varied among the 282 cities, ranging from 6 to 114 μg/m^3^ (mean, 52.6 μg/m^3^). Each 10-mg/m^3^ increase in the PM_2.5_ concentration was associated with 14% higher odds for membranous nephropathy^9^ (odds ratio, 1.14; 95% confidence interval, 1.10–1.18) in regions with PM_2.5_ concentration in excess of 70 μg/m^3^.

### Strengths and limitations

In our current study, we related the renal health outcomes of individual study participants with subject-level estimates of exposure to ultrafine particulate matter. This approach mitigates the so-called ecological fallacy, which arises when aggregate measures of outcome, e.g., cancer incidence by municipality are associated with aggregate estimates of exposure^18^, an approach applied in several previous studies of the renal responses to exposure to PM2.5. Our study is the first to address renal outcomes in relation to BC. Furthermore, our cohort study was population based and therefore representative for exposure, housing conditions and lifestyle in an affluent West-European country. Our study included cross-sectional and longitudinal analyses, both adjusted for a wide range of confounders, in the longitudinal analyses in a time-dependent manner. Sensitivity analyses stratified for various subgroups and minimally adjusted analyses were confirmatory. Notwithstanding these strong points, our study must also be interpreted within the context of its potential limitations. First, the sample size of the study population was relatively small compared with other studies of renal function in relation to PM2.5^9,10^. Second, due to a change in methodology, serum creatinine was measured by slightly different methods in 536 of 653 participants (82.1%) at baseline and follow-up. However, as reported previously^19^, the difference in the annual decrease in eGFR between participants who had their serum creatinine measured by different compared with the same method averaged only 0.05 mL/min/1.73 m^2^ (*P* = 0.60). Moreover, whatever creatinine assay was used, the annual eGFR decrease was similar as that observed in other European population studies^20^. Third, as in previous studies of chronic air pollution^21^, the assessment of renal function and the collection of the air pollution data was not done simultaneously. On the other hand, we used high-resolution spatial data. Several studies in the Netherlands^22^, Italy (Rome)^23^, the UK^24^, and Canada (Vancouver)^25^ demonstrated that the land use models applied in our current study are representative for the air pollution over periods of 10 years or longer prior to the actual modelling^22–25^. Fourth, the incidence of microalbuminuria was low, precluding subgroup analyses for this endpoint. The data given in Table 5 including all data available in the whole study cohort are therefore only given as an exploratory result. Finally, the prevalence of previous cardiovascular disease and diabetes in our study participants was low, precluding extrapolation of our current findings to patients with comorbidities, who might be more vulnerable with regard to the renal responses to exposure to ultrafine toxic particulate.

## Methods

### Study population

The Flemish Study on Environment, Genes and Health Outcomes (FLEMENGHO) complies with the Helsinki declaration^26^ and is registered at the Belgian Data Protection Authority (reference number III 11/1234/13, dated 22 August 2013). The ethics committee of the University Hospital Leuven, Belgium, approved secondary use of FLEMENGHO data (national registration number, B32220083510). From August 1985 until November 1990, a random sample of the households, stratified for sex and age (20–39, 40–59, and ≥ 60 years) and living in a geographically defined area of Northern Belgium (Figs. 1, 2) was investigated. All household members with a minimum age of 20 years were invited to take part. From June 1996 until January 2004 recruitment of families continued, using the former participants (1985 − 1990) as index persons and also including teenagers^13,27^. The initial participation rate at enrolment was 78%. The informed consent was obtained from all participants.

Participants were repeatedly followed-up. From 2005 to 2009, we invited 1031 surviving participants, who still resided in the catchment area of the study (North Limburg, Belgium) and who had not been institutionalised. Of these, 828 renewed informed consent (participation rate, 80.3%). We excluded 8 participants, because their renal function had not been assessed. Of 820 participants examined at baseline, 653 took part in a follow**-**up assessment of their renal function on average 4.7 years (5th–95th percentile, 3.7–5.4 years) later^13,19,27^. Therefore, the number of participants available for the cross-sectional and longitudinal analyses totalled 820 and 653, respectively.

### Clinical measurements

Blood pressure (continuous) was the average of five consecutive auscultatory readings obtained after participants had rested for 5 min in the sitting position. Mean arterial pressure (continuous) was diastolic blood pressure plus one third of pulse pressure (the difference between systolic and diastolic blood pressure). Hypertension (categorical) was a blood pressure of ≥ 140 mm Hg systolic, or ≥ 90 mm Hg diastolic, or use of antihypertensive drugs. Antihypertensive drugs were classified (categorical) as diuretics (thiazides, loop diuretics and aldosterone antagonists), inhibitors of the renin-angiotensin system (β-blockers, angiotensin-converting enzyme inhibitors and angiotensin type-1 receptor antagonists), and vasodilators (calcium-channel blockers and α-blockers). Body mass index (continuous) was weight (kilogram) divided by height (metre) squared.

The study nurses also administered a standardised questionnaire inquiring into each participant’s medical history, smoking and drinking habits, intake of medications, habitual physical activity during leisure time and at work, and socioeconomic status. Smoking was the current use of any smoking materials on a daily basis (categorical). The amount of alcohol consumed expressed in gram per day was computed from the self-reported frequency and type of alcoholic beverages used (beer, wine, fortified wine and liquor) and their alcohol content (6, 13, 18 and 40 volumetric percentages, respectively)^28^. Participants with a daily alcohol consumption of 5 g/day or more were categorised as drinkers.

Socioeconomic status was coded according to the complex scales provided by the UK Office of Population Censuses and Surveys^29^, and simplified into a linear scale with scores ranging from 1 to 3^30^. Using validated questionnaires^28^ and published tables^31^, we computed the energy spent in physical activity (continuous) from body weight, time devoted to work, walking, sports and leisure time activities, and type of physical activity. This covariable was introduced, because of the known associations of eGFR^32,33^ and albuminuria^32,34^ with strenuous physical activity.

### Biochemical measurements

Venous blood samples were obtained after 8 h fasting. Participants collected a 24-h urine sample. After centrifugation and aliquoting, blood-derived specimens were stored at − 80 °C and 10-ml urine samples at − 40 °C. A single certified laboratory did the routine biochemistry, using quality-controlled automated methods. Blood samples were analysed for plasma glucose and total and high-density lipoprotein (HDL) serum cholesterol, serum creatinine and serum γ**-**glutamyltransferase (biomarker of alcohol intake). Diabetes mellitus was a fasting glucose exceeding 7.0 mmol/L (126 mg/dL) or use of antidiabetic agents. eGFR was calculated based on the Chronic Kidney Disease Epidemiology Collaboration (CKD-EPI) equation^35^, using age, sex and serum creatinine. The single laboratory involved in measuring the serum creatinine concentration in our study implemented the IDMS**-**traceable (isotope**-**dilution mass spectrometry) creatinine assay^36^, starting from 18 December 2008. Of the baseline creatinine measurements 703 (85.7%) were done before this date. The remaining 117 baseline measurements and all 653 follow-up measurements were done after 18 December 2008. The urine samples were analysed for microalbumin and creatinine. CKD was staged following the National Kidney Foundation (KDOQI) guideline^37^. Microalbuminuria was defined by an albumin-to-creatinine ratio of at least 3.5 mg/mmol in women or 2.5 mg/mmol in men^38^. A single blood and urine sample were used to determine the CKD endpoint in categorical analyses, defined as an eGFR of 60 mL/min/1.73 m^2^, in line with our previous research^19,27^ and landmark population studies^39,40^.

### Ambient air pollution

Particulate matter is the sum of all solid and liquid particles suspended in air^17^. PM_2.5_ has an aerodynamic diameter of less than 2.5 μm. Upon inhalation, PM_2.5_ reaches the smallest airways and alveoli and can even cross the blood-air barrier and directly penetrate into the blood stream. BC is a component of PM_2.5_, consists of pure carbon in several bond forms, and finds its origin in the incomplete combustion of fossil fuels, biofuel or biomass.

In the current study, BC and PM_2.5_ exposure (µg/m^3^) were calculated for each participant’s residential address at the time of the air quality measurements (2010–2014), using a high resolution spatiotemporal interpolation method^41,42^ that takes into account land-cover data obtained from satellite images (CORINE database [http://www.eea.europa.eu/publications/COR0-landcover/at_download/file]) and pollution data of fixed monitoring stations in combination with a dispersion model^41,42^. This approach provides daily exposure values in a dense irregular receptor grid, using input from the Belgian telemetric air quality networks and emissions from point and line sources. The air quality network includes 14 monitoring stations for black carbon and 34 for PM_2.5_. Models covered data averaged over a 5-year period (2010–2014), which reflect the long-term spatial air pollution and are representative for earlier periods^22–25^. Model performance was evaluated by leave-one-out cross-validation across monitoring stations. The spatiotemporal explained variance was nearly 80%^41,42^ for BC and PM_2.5_.

### Statistical analysis

For database management and statistical analysis, we used the SAS system, version 9.4 (SAS Institute Inc., Cary, NC). Departure from normality was evaluated by Shapiro–Wilk’s statistic. We normalised the distributions of γ-glutamyltransferase, and the daily energy expenditure in physical activity by a logarithmic transformation. In between-group analyses, means were compared using the large-sample z-test, ANOVA and proportions by Fisher’s exact test. Longitudinal changes were assessed by a t-statistic of the distributions of the follow-up minus baseline values for continuous variables and by the McNemar test for categorical variables. We rank normalised the distribution of BC and PM_2.5_ by sorting the measurements from the smallest to the largest and then applying the inverse cumulative normal function. We computed Pearson correlation coefficients between continuously distributed data and Spearman rank correlations to assess the association between geocorrelation between percentage changes in eGFR and the exposure to BC and PM2.5.

For the cross-sectional analysis, the association of renal function measures with air pollution was assessed by a generalised linear mixed model adjusting for residential clustering of participants (sharing the same address) as random effect and with adjustments applied for relevant covariables^43,44^. The covariables in the cross-sectional analyses included: sex, age (linear and squared term), mean arterial pressure, heart rate, body mass index, plasma glucose, total-to-HDL cholesterol ratio, γ-glutamyltransferase as biomarker of alcohol consumption, smoking, daily energy expenditure in physical activity, socioeconomic class, and antihypertensive treatment (by drug class). In longitudinal analyses of 653 participants, we accounted for baseline serum creatinine and baseline eGFR by calculating the percent change between the repeat and the first measurement of these biomarkers, while adjusting for sex, baseline age (linear and square term), socioeconomic class, follow-up duration, baseline body mass index, and the baseline value of and change during follow-up in mean arterial pressure, heart rate, plasma glucose, the total-to-HDL serum cholesterol ratio, γ-glutamyltransferase, smoking status, and the intake of antihypertensive drugs (all drugs combined). The association of the prevalence (cross-sectional analyses) or incidence (longitudinal analyses) of CKD and microalbuminuria with the exposure to particulate were derived by mixed models and expressed as odds ratios, adjusted as outlined above.

In sensitivity analyses, we stratified the participants according to sex, age (< 60 vs. ≥ 60 years), smoking status (non-smokers versus smokers), and the amount of alcohol consumed per day (< 5 vs. ≥ 5 g). In addition, we re-ran the cross-sectional and longitudinal analyses, while adjusting for a reduced set of covariables, thereby removing potential mediators of renal function decline.

### Ethical approval

The Flemish Study on Environment, Genes and Health Outcomes complies with the Helsinki declaration and is registered at the Belgian Data Protection Authority (reference number III 11/1234/13, dated 22 August 2013). The ethics committee of the University Hospital Leuven, Belgium, approved the study (national registration number: B32220083510).

### Data sharing

Anonymised participants data will be made available upon request directed to the corresponding author. Proposals will be reviewed and approved by the authors with scientific merit and feasibility as sole criteria. After approval of a proposal, data can be shared via a secure online platform after signing a data access and confidentiality agreement. All data will be made available for a minimum of 5 years from the end of the study.

## Conclusions

Exposure to polluted air, in contrast to smoking, is unintentional and adversely affects health in multiple ways, so that prevention is of paramount importance and global efforts to improve air quality should continue. What our current study adds is that at current exposure levels in a semirural area of Flanders renal function is not associated with ultrafine particulate, including BC, in predominantly healthy people representative of the general population.

## Supplementary Information

Supplementary Information.

## Abbreviations

BC: Black carbon
FLEMENGHO: Flemish Study on Environment, Genes and Health Outcomes
CKD-EPI: Chronic Kidney Disease Epidemiology Collaboration
CKD: Chronic kidney disease
eGFR: Estimated glomerular filtration rate derived from serum creatinine
