# Supplementary material for: Glomerular function in relation to fine airborne particulate matter in a representative population sample
Source: Sci Rep. 2021 Jul 19;11:14646. doi: 10.1038/s41598-021-94136-1 (PMC8290004; doi:10.1038/s41598-021-94136-1)
Supplement: Supplementary file 1 — Supplementary Information. [file 41598_2021_94136_MOESM1_ESM.docx]

**Scientific Reports**

**SUPPLEMENTARY MATERIAL**

This web appendix formed part of the original submission and has been peer reviewed.
Supplement to: ***Renal function in relation to fine airborne particulate matter in a representative population sample***

*Ying-Mei Feng, Lutgarde Thijs, Zhen-Yu Zhang, Esmée M. Bijnens, Wen-Yi Yang, Fang-Fei Wei, Bram G. Janssen, Tim S Nawrot, Jan A. Staessen*

**Table of contents**

**Table S1** Correlation matrix of baseline covariables in men p2

**Table S2** Correlation matrix of baseline covariables in women p3

**Table S3** Baseline characteristics of participants by sex p4

**Table S4** Baseline characteristics of participants by age p5

**Table S5** Baseline characteristics of participants by smoking status p6

**Table S6** Baseline characteristics of participants by daily alcohol intake p7

**Table S7** Multivariable-adjusted cross-sectional associations of renal function with black carbon
in various subgroups p8

**Table S8** Multivariable-adjusted cross-sectional associations of renal function with PM2.5
in various subgroups p9

**Table S9** Multivariable-adjusted between changes from baseline to follow-up in renal function
and in the black carbon exposure in various subgroups p10

**Table S10** Multivariable-adjusted between changes from baseline to follow-up in renal function
and in the PM2.5 exposure in various subgroups p11

**Table S11** Cross-sectional associations of renal function with black carbon and PM2.5 with a
reduced set of covariables p12

**Table S12** Longitudinal associations of renal function with black carbon and PM2.5 with a
reduced set of covariables p13

**Figure S1** Distribution plots serum creatinine and eGFR by sex and age p14

**Figure S2** Association of serum creatinine and eGFR with age by sex p15

**Supplementary Table 1**

**. Correlation coefficients between continuously distributed baseline covariables in 404 men.**

|  | SCRT | eGFR | Age | SBP | DBP | MAP | HR | BMI | GLC | TC | HC | THCR | LGGT | LTCAL | SOC |
| --- | --- | --- | --- | --- | --- | --- | --- | --- | --- | --- | --- | --- | --- | --- | --- |
| SCRT | ― |  |  |  |  |  |  |  |  |  |  |  |  |  |  |
| eGFR | -0.75§ | ― |  |  |  |  |  |  |  |  |  |  |  |  |  |
| Age | 0.20§ | -0.72§ | ― |  |  |  |  |  |  |  |  |  |  |  |  |
| SBP | 0.17‡ | -0.37§ | 0.43§ | ― |  |  |  |  |  |  |  |  |  |  |  |
| DBP | 0.08 | -0.18‡ | 0.13* | 0.49§ | ― |  |  |  |  |  |  |  |  |  |  |
| MAP | 0.13† | -0.30§ | 0.30§ | 0.83§ | 0.89 | ― |  |  |  |  |  |  |  |  |  |
| HR | -0.05 | 0.04 | -0.01 | 0.13† | 0.23 | 0.22§ | ― |  |  |  |  |  |  |  |  |
| BMI | 0.16† | 0.31§ | 0.31§ | 0.21§ | 0.31 | 0.31§ | 0.07 | ― |  |  |  |  |  |  |  |
| GLC | 0.04 | -0.17‡ | 0.20§ | 0.24§ | -0.01 | 0.12* | 0.07 | 0.18‡ | ― |  |  |  |  |  |  |
| TC | 0.02 | -0.14† | 0.18‡ | 0.16† | 0.28 | 0.27§ | 0.13† | 0.20§ | 0.004 | ― |  |  |  |  |  |
| HC | -0.15† | 0.15† | -0.09 | -0.003 | 0.07 | 0.04 | 0.04 | -0.22§ | -0.10* | 0.27§ | ― |  |  |  |  |
| THCR | 0.15† | -0.24§ | 0.23§ | 0.12* | 0.12 | 0.14† | 0.08 | 0.35§ | 0.11* | 0.49§ | -0.68§ | ― |  |  |  |
| LGGT | 0.12* | -0.13 | 0.09 | 0.17‡ | 0.26 | 0.26§ | 0.17‡ | 0.32§ | 0.07 | 0.30§ | -0.06 | 0.27§ | ― |  |  |
| LTCAL | -0.01 | 0.02 | -0.01 | -0.01 | 0.10 | 0.06 | 0.07 | 0.14† | 0.04 | 0.01 | 0.03 | -0.02 | -0.02 | ― |  |
| SOC | -0.06 | 0.10* | -0.21§ | -0.13† | 0.10* | -0.004 | -0.08 | -0.08 | -0.11* | 0.06 | 0.12* | -0.08 | -0.03 | -0.24§ | ― |

Abbreviation: SCR, serum creatinine concentration; eGFR, glomerular filtration rate estimated from serum creatinine; SBP, systolic blood pressure; DBP, diastolic blood pressure; MAP, mean arterial pressure; HR, heart rate; BMI, body mass index; GLC, plasma glucose; TC, total serum cholesterol, HC, high-density lipoprotein serum cholesterol; THCR, total-to-high-density lipoprotein serum cholesterol; LGGT, serum γ‑glutamyltransferase; LTCAL, total daily energy expenditure in physical activity (logarithmically transformed); SEC, socioeconomic class. * P ≤ 0.05; † P ≤ 0.01; ‡ P ≤ 0.001; § *P ≤* 0.0001.

**Supplementary Table 2**

**. Correlation coefficients between continuously distributed baseline covariables in 416 women.**

|  | SCRT | eGFR | Age | SBP | DBP | MAP | HR | BMI | GLC | TC | HC | THCR | LGGT | LTCAL | SOC |
| --- | --- | --- | --- | --- | --- | --- | --- | --- | --- | --- | --- | --- | --- | --- | --- |
| SCRT | ― |  |  |  |  |  |  |  |  |  |  |  |  |  |  |
| eGFR | -0.76§ | ― |  |  |  |  |  |  |  |  |  |  |  |  |  |
| Age | 0.18‡ | -0.69§ | ― |  |  |  |  |  |  |  |  |  |  |  |  |
| SBP | 0.21§ | -0.46§ | 0.60§ | ― |  |  |  |  |  |  |  |  |  |  |  |
| DBP | 0.11§ | -0.23§ | 0.23§ | 0.54§ | ― |  |  |  |  |  |  |  |  |  |  |
| MAP | 0.19§ | -0.40§ | 0.48§ | 0.88§ | 0.87§ | ― |  |  |  |  |  |  |  |  |  |
| HR | 0.02 | 0.07 | -0.14† | -0.02 | 0.20§ | 0.10* | ― |  |  |  |  |  |  |  |  |
| BMI | 0.15† | -0.21§ | 0.21§ | 0.32§ | 0.31§ | 0.36§ | 0.17‡ | ― |  |  |  |  |  |  |  |
| GLC | 0.13† | -0.30§ | 0.37§ | 0.25§ | 0.12* | 0.22§ | 0.05 | 0.17‡ | ― |  |  |  |  |  |  |
| TC | 0.12* | -0.26§ | 0.30§ | 0.27§ | 0.17§ | 0.25§ | -0.004 | 0.16† | 0.09 | ― |  |  |  |  |  |
| HC | -0.09 | 0.10 | -0.04 | -0.03 | -0.06 | -0.05 | -0.06 | -0.28§ | -0.15† | 0.27§ | ― |  |  |  |  |
| THCR | 0.18‡ | -0.28§ | 0.25§ | 0.23§ | 0.19§ | 0.24§ | 0.07 | 0.37§ | 0.21§ | 0.51§ | -0.66§ | ― |  |  |  |
| LGGT | 0.04 | -0.20§ | 0.29§ | 0.27§ | 0.18§ | 0.26§ | 0.09 | 0.18‡ | 0.29§ | 0.16§ | -0.09 | 0.22§ | ― |  |  |
| LTCAL | 0.02 | -0.16† | 0.29§ | 0.21§ | 0.10§ | 0.18‡ | -0.05 | 0.15† | 0.09 | 0.09 | 0.09 | 0.06 | 0.11* | ― |  |
| SOC | -0.002 | 0.07 | -0.17‡ | -0.16† | 0.01 | -0.09 | -0.03 | -0.15† | -0.08 | -0.05 | -0.05 | -0.12* | -0.09 | -0.25* | ― |

Abbreviation: SCR, serum creatinine concentration; eGFR, glomerular filtration rate estimated from serum creatinine; SBP, systolic blood pressure; DBP, diastolic blood pressure; MAP, mean arterial pressure; HR, heart rate; BMI, body mass index; GLC, plasma glucose; TC, total serum cholesterol, HC, high-density lipoprotein serum cholesterol; THCR, total-to-high-density lipoprotein serum cholesterol; LGGT, serum γ‑glutamyltransferase; LTCAL, total daily energy expenditure in physical activity (logarithmically transformed); SEC, socioeconomic class. * P ≤ 0.05; † P ≤ 0.01; ‡ P ≤ 0.001; § P *≤* 0.0001.

**Supplementary Table 3.**

**Baseline characteristics of participants by sex.**

| **Characteristic** | **Men** |  | **Women** | **P** |
| --- | --- | --- | --- | --- |
| All in category (n) | 404 |  | 416 |  |
| Smokers (n [%]) | 85 (21.0) |  | 83 (20.0) | 0.70 |
| Alcohol intake ≥5g/day (n [%]) |  |  |  |  |
| Hypertension (n [%]) | 179 (44.3) |  | 162 (38.9) | 0.12 |
| Antihypertensive treatment (n [%]) | 101 (25.0) |  | 110 (26.4) | 0.64 |
| Statin treatment (n [%]) | 50 (12.4) |  | 56 (13.5) | 0.64 |
| Diabetes mellitus (n [%]) | 18 (4.5) |  | 14 (3.4) | 0.42 |
| Microalbuminuria (n [%]) | 308 (80.6) |  | 315 (79.2) | 0.61 |
| CKD, stage ≥3 (n [%]) | 25 (6.2) |  | 44 (10.6) | 0.0006 |
| Mean of characteristic |  |  |  |  |
| Age (years) | 50.7 (16.1) |  | 51.6 (15.2) | 0.40 |
| Body mass index (kg/m2) | 26.7 (3.9) |  | 26.3 (4.7) | 0.15 |
| Systolic pressure (mm Hg) | 131.0 (15.4) |  | 127.8 (19.2) | 0.008 |
| Diastolic pressure (mm Hg) | 81.6 (9.6) |  | 77.8 (9.1) | <0.0001 |
| Mean arterial pressure (mm Hg) | 98.1 (10.0) |  | 94.5 (10.9) | <0.0001 |
| Heart rate (beats per minute) | 62.1 (9.7) |  | 64.8 (9.6) | <0.0001 |
| Biochemical data |  |  |  |  |
| Serum creatinine (μmol/L) | 94.2 (15.3) |  | 86.2 (13.5) | <0.0001 |
| eGFR (mL/min/1.73 m2) | 83.8 (16.6) |  | 78.1 (15.7) | <0.0001 |
| Total cholesterol (mmol/L) | 5.12 (0.94) |  | 5.39 (0.98) | <0.0001 |
| HDL cholesterol (mmol/L) | 1.27 (0.28) |  | 1.57 (0.34) | <0.0001 |
| Total-to-HDL cholesterol ratio | 4.19 (1.06) |  | 3.55 (0.90) | <0.0001 |
| Plasma glucose (mmol/L) | 5.05 (0.96) |  | 4.84 (0.61) | 0.0002 |
| γ-glutamyltransferase (U/L) | 35 (13–89) |  | 24 (9–64) | <0.0001 |
| Airborne particulate |  |  |  |  |
| Black carbon (μg/m3) | 1.17 (0.20) |  | 1.17 (0.20) | 0.69 |
| PM2.5 (μg/m3) | 13.5 (0.92) |  | 13.5 (0.91) | 0.57 |

Abbreviations: CKD, chronic kidney disease; eGFR, glomerular filtration rate estimated from serum creatinine by the CKD-EPI formula; HDL, high-density lipoprotein. Blood pressure was the average of five consecutive auscultatory readings. Hypertension was a blood pressure of ≥140 mm Hg systolic or ≥90 mm Hg diastolic, or use of antihypertensive drugs. Diabetes mellitus was a fasting plasma glucose >7.0 mmol/L (>126 mg/dL) or use of antidiabetic agents. The assessment of microalbuminuria (>3.5/>2.5 mg per mmol creatinine in women/men) was available in 780 participants. The central tendency (data spread) is given as arithmetic mean (SD) or median (5th-5th percentile interval). P‑values indicate the significance of the sex differences.

**Supplementary Table 4.**

**Baseline characteristics of participants by age.**

| **Characteristic** | **<60 years** |  | **≥60years** | **P** |
| --- | --- | --- | --- | --- |
| All in category (n) | 588 |  | 232 |  |
| Women (n [%]) | 297 (50.5) |  | 119 (51.3) | 0.84 |
| Alcohol intake ≥5g/day (n [%]) | 265 (45.1) |  | 67 (28.9) | <0.0001 |
| Hypertension (n [%]) | 177 (30.1) |  | 164 (70.7) | <0.0001 |
| Antihypertensive treatment (n [%]) | 97 (16.5) |  | 114 (49.1) | <0.0001 |
| Statin treatment (n [%]) | 48 (8.2) |  | 58 (25.0) | <0.0001 |
| Diabetes mellitus (n [%]) | 12 (2.0) |  | 20 (8.6) | <0.0001 |
| Microalbuminuria (n [%]) | 14 (2.5) |  | 15 (6.9) | 0.004 |
| CKD, stage ≥3 (n [%]) | 11 (1.9) |  | 68 (29.3) | <0.0001 |
| Mean of characteristic |  |  |  |  |
| Age (years) | 43.7 (11.5) |  | 69.9 (6.3) | 0.04 |
| Body mass index (kg/m2) | 26.1 (4.4) |  | 27.6 (4.1) | <0.0001 |
| Systolic pressure (mm Hg) | 124.4 (13.7) |  | 142.1 (19.4) | 0.004 |
| Diastolic pressure (mm Hg) | 80.0 (9.7) |  | 79.0 (9.0) | 0.002 |
| Mean arterial pressure (mm Hg) | 94.8 (10.3) |  | 100.0 (10.6) | 0.0005 |
| Heart rate (beats per minute) | 64.1 (9.5) |  | 61.9 (10.0) | 0.53 |
| Biochemical data |  |  |  |  |
| Serum creatinine (μmol/L) | 84.8 (12.4) |  | 90.6 (20.9) | 0.0001 |
| eGFR (mL/min/1.73 m2) | 86.3 (14.4) |  | 67.1 (12.7) | <0.0001 |
| Total cholesterol (mmol/L) | 5.21 (0.97) |  | 5.38 (0.94) | 0.02 |
| HDL cholesterol (mmol/L) | 1.43 (0.35) |  | 1.40 (0.35) | 0.18 |
| Total-to-HDL cholesterol ratio | 3.80 (1.02) |  | 4.03 (1.06) | 0.003 |
| Plasma glucose (mmol/L) | 4.84 (0.61) |  | 5.21 (1.13) | <0.0001 |
| γ-glutamyltransferase (U/L) | 29 (10–71) |  | 32 (12–67) | 0.36 |
| Airborne particulate |  |  |  |  |
| Black carbon (μg/m3) | 1.19 (0.20) |  | 1.13 (0.17) | <0.0001 |
| PM2.5 (μg/m3) | 13.6 (0.95) |  | 13.3 (0.79) | <0.0001 |

Abbreviations: CKD, chronic kidney disease; eGFR, glomerular filtration rate estimated from serum creatinine by the CKD-EPI formula; HDL, high-density lipoprotein. Blood pressure was the average of five consecutive auscultatory readings. Hypertension was a blood pressure of ≥140 mm Hg systolic or ≥90 mm Hg diastolic, or use of antihypertensive drugs. Diabetes mellitus was a fasting plasma glucose of >7.0 mmol/L (>126 mg/dL) or use of antidiabetic agents. The assessment of microalbuminuria (>3.5/>2.5 mg per mmol creatinine in women/men) was available in 780 participants. The central tendency (data spread) is given as arithmetic mean (SD) or median (5th-5th percentile interval). P‑values indicate the significance of the differences between younger and older participants.

**Supplementary Table 5.**

**Baseline characteristics of participants by smoking status.**

| **Characteristic** | **Non-smokers** |  | **Smokers** | **P** |
| --- | --- | --- | --- | --- |
| All in category (n) | 652 |  | 168 |  |
| Women (n [%]) | 333 (51.1) |  | 83 (49.4) | 0.70 |
| Alcohol intake ≥5g/day (n [%]) |  |  |  |  |
| Hypertension (n [%]) | 292 (44.8) |  | 49 (29.2) | 0.002 |
| Antihypertensive treatment (n [%]) | 181 (27.8) |  | 30 (17.9) | 0.009 |
| Statin treatment (n [%]) | 93 (14.3) |  | 13 (7.7) | 0.02 |
| Diabetes mellitus (n [%]) | 28 (4.3) |  | 4 (2.4) | 0.25 |
| Microalbuminuria (n [%]) | 507 (81.1) |  | 116 (74.8) | 0.08 |
| CKD, stage ≥3 (n [%]) | 60 (6.2) |  | 9 (10.6) | 0.12 |
| Mean of characteristic |  |  |  |  |
| Age (years) | 51.7 (15.9) |  | 48.9 (14.3) | 0.04 |
| Body mass index (kg/m2) | 26.8 (4.4) |  | 25.3 (4.0) | <0.0001 |
| Systolic pressure (mm Hg) | 130.3 (17.8) |  | 125.9 (15.7) | 0.004 |
| Diastolic pressure (mm Hg) | 80.2 (9.6) |  | 77.6 (8.8) | 0.002 |
| Mean arterial pressure (mm Hg) | 96.9 (10.7) |  | 93.7 (9.7) | 0.0005 |
| Heart rate (beats per minute) | 63.4 (9.8) |  | 63.9 (9.5) | 0.53 |
| Biochemical data |  |  |  |  |
| Serum creatinine (μmol/L) | 87.2 (16.3) |  | 83.6 (11.5) | 0.007 |
| eGFR (mL/min/1.73 m2) | 79.9 (16.5) |  | 84.9 (15.5) | 0.0004 |
| Total cholesterol (mmol/L) | 5.25 (0.97) |  | 5.25 (0.95) | 0.96 |
| HDL cholesterol (mmol/L) | 1.43 (0.35) |  | 1.41 (0.35) | 0.52 |
| Total-to-HDL cholesterol ratio | 3.85 (1.02) |  | 3.91 (1.09) | 0.49 |
| Plasma glucose (mmol/L) | 4.96 (0.86) |  | 4.86 (0.55) | 0.13 |
| γ-glutamyltransferase (U/L) | 29 (10–66) |  | 32 (11–76) | 0.33 |
| Airborne particulate |  |  |  |  |
| Black carbon (μg/m3) | 1.16 (0.19) |  | 1.21 (0.21) | 0.004 |
| PM2.5 (μg/m3) | 13.4 (0.90) |  | 13.6 (0.96) | 0.02 |

Abbreviations: CKD, chronic kidney disease; eGFR, glomerular filtration rate estimated from serum creatinine by the CKD-EPI formula; HDL, high-density lipoprotein. Blood pressure was the average of five consecutive auscultatory readings. Hypertension was a blood pressure of ≥140 mm Hg systolic or ≥90 mm Hg diastolic, or use of antihypertensive drugs. Diabetes mellitus was a fasting plasma glucose of >7.0 mmol/L (>126 mg/dL) or use of antidiabetic agents. The assessment of microalbuminuria (>3.5/>2.5 mg per mmol creatinine in women/men) was available in 780 participants. The central tendency (data spread) is given as arithmetic mean (SD) or median (5th-5th percentile interval). P‑values indicate the significance of the differences between non-smokers and smokers.

**Supplementary Table 6.**

**Baseline characteristics of participants by daily alcohol intake.**

| **Characteristic** | **<5g/day** |  | **≥5 g/day** | **P** |
| --- | --- | --- | --- | --- |
| All in category (n) | 488 |  | 332 |  |
| Women (n [%]) | 325 (66.6) |  | 91 (27.4) | <0.0001 |
| Smokers (n [%]) | 88 (18.0) |  | 80 (24.1) | 0.03 |
| Hypertension (n [%]) | 210 (43.0) |  | 131 (39.5) | 0.31 |
| Antihypertensive treatment (n [%]) | 135 (27.7) |  | 76 (22.9) | 0.12 |
| Statin treatment (n [%]) | 69 (14.1) |  | 37 (11.1) | 0.21 |
| Diabetes mellitus (n [%]) | 27 (5.5) |  | 5 (1.5) | 0.004 |
| Microalbuminuria (n [%]) | 360 (77.1) |  | 263 (84.0) | 0.02 |
| CKD, stage ≥3 (n [%]) | 57 (6.2) |  | 12 (10.6) | <0.0001 |
| Mean of characteristic |  |  |  |  |
| Age (years) | 53.2 (15.8) |  | 48.2 (14.9) | 0.04 |
| Body mass index (kg/m2) | 26.7 (4.6) |  | 26.2 (4.0) | <0.0001 |
| Systolic pressure (mm Hg) | 129.7 (18.2) |  | 129.0 (16.4) | 0.004 |
| Diastolic pressure (mm Hg) | 79.0 (9.3) |  | 80.7 (9.7) | 0.002 |
| Mean arterial pressure (mm Hg) | 95.9 (10.6) |  | 96.8 (10.7) | 0.0005 |
| Heart rate (beats per minute) | 64.0 (9.6) |  | 62.7 (9.8) | 0.53 |
| Biochemical data |  |  |  |  |
| Serum creatinine (μmol/L) | 84.7 (16.9) |  | 88.9 (12.8) | <0.0001 |
| eGFR (mL/min/1.73 m2) | 78.5 (16.2) |  | 84.5 (16.1) | <0.0001 |
| Total cholesterol (mmol/L) | 5.29 (0.97) |  | 5.19 (0.96) | 0.15 |
| HDL cholesterol (mmol/L) | 1.45 (0.36) |  | 1.39 (0.33) | 0.01 |
| Total-to-HDL cholesterol ratio | 3.83 (1.01) |  | 3.92 (1.06) | 0.21 |
| Plasma glucose (mmol/L) | 4.97 (0.92) |  | 4.90 (0.61) | 0.21 |
| γ-glutamyltransferase (U/L) | 27 (10–64) |  | 33 (11–81) | 0.009 |
| Airborne particulate |  |  |  |  |
| Black carbon (μg/m3) | 1.17 (0.20) |  | 1.16 (0.19) | 0.61 |
| PM2.5 (μg/m3) | 13.5 (0.92) |  | 13.5 (0.91) | 0.87 |

Abbreviations: CKD, chronic kidney disease; eGFR, glomerular filtration rate estimated from serum creatinine by the CKD-EPI formula; HDL, high-density lipoprotein. Blood pressure was the average of five consecutive auscultatory readings. Hypertension was a blood pressure of ≥140 mm Hg systolic or ≥90 mm Hg diastolic, or use of antihypertensive drugs. Diabetes mellitus was a fasting plasma glucose of >7.0 mmol/L (>126 mg/dL) or use of antidiabetic agents. The assessment of microalbuminuria (>3.5/>2.5 mg per mmol creatinine in women/men) was available in 780 participants. The central tendency (data spread) is given as arithmetic mean (SD) or median (5th-5th percentile interval). P‑values indicate the significance of the differences between participants dichotomised by a daily alcohol of 5 g per day.

**Supplementary Table 7.**

**Multivariable-adjusted cross-sectional associations of renal function with black carbon in various subgroups.**

| **Group analysed   Renal function trait** |  | **Estimate (95% CI)** | **P** |  | **Estimate (95% CI)** | **P** |  | **Pint** |
| --- | --- | --- | --- | --- | --- | --- | --- | --- |
| ***Sex*** |  | ***Women (N = 416)*** | |  | ***Men (N = 404)*** | |  |  |
| Serum creatinine (μmol/L) |  | 0.17 (-1.00, 1.34) | 0.77 |  | 0.11 (-1.38, 1.60) | 0.89 |  | 0.45 |
| eGFR (mL/min/1.73 m2) |  | 0.04 (-1.11, 1.19) | 0.94 |  | 0.15 (-1.03, 1.34) | 0.80 |  | 0.79 |
| Prevalence |  |  |  |  |  |  |  |  |
| Chronic kidney disease (OR) |  | -0.12 (-0.58, 0.33) | 0.60 |  | -0.25 (-0.80, 0.31) | 0.38 |  | 0.76 |
| Microalbuminuria (OR) |  | -0.02 (-0.27, 0.24) | 0.72 |  | 0.12 (-0.17, 0.40) | 0.42 |  | 0.87 |
| ***Age*** |  | ***<60 years (N = 588)*** | |  | ***≥60 years (N = 232)*** | |  |  |
| Serum creatinine (μmol/L) |  | 0.32 (-0.60, 1.23) | 0.50 |  | 0.27 (-2.21, 2.74) | 0.83 |  | 0.70 |
| eGFR (mL/min/1.73 m2) |  | -0.03 (-1.03, 0.97) | 0.96 |  | -0.22 (-1.81, 1.38) | 0.79 |  | 0.48 |
| Prevalence |  |  |  |  |  |  |  |  |
| Chronic kidney disease (OR) |  | -0.039 (…) | … |  | -0.16 (-0.55, 0.24) | 0.44 |  | 0.94 |
| Microalbuminuria (OR) |  | -0.02 (-0.25, 0.22) | 0.89 |  | 0.34 (-0.06, 0.75) | 0.10 |  | 0.12 |
| ***Smoking status*** |  | ***Non-smokers*** ***(N = 685)*** | |  | ***Smokers (N = 135)*** | |  |  |
| Serum creatinine (μmol/L) |  | 0.49 (-0.63, 1.61) | 0.39 |  | -0.52 (-1.93, 0.89) | 0.48 |  | 0.36 |
| eGFR (mL/min/1.73 m2) |  | -0.36 (-1.36, 0.64) | 0.47 |  | 0.81 (-0.82, 2.43) | 0.34 |  | 0.49 |
| Prevalence |  |  |  |  |  |  |  |  |
| Chronic kidney disease (OR) |  | -0.06 (-0.41, 0.29) | 0.73 |  | -0.94 | NA |  | 0.71 |
| Microalbuminuria (OR) |  | 0.09 (-0.13, 0.31 | 0.43 |  | -0.26 (-0.57, 0.05) | 0.10 |  | 0.75 |
| ***Alcohol consumption*** |  | ***<5 g/day (N = 350)*** | |  | ***≥5 g/day (N = 470)*** | |  |  |
| Serum creatinine (μmol/L) |  | -0.06 (-1.37, 1.25) | 0.93 |  | 0.89 (-0.36, 2.14) | 0.17 |  | 0.69 |
| eGFR (mL/min/1.73 m2) |  | 0.18 (-0.94, 1.30) | 0.57 |  | -0.77 (-2.10, 0.56) | 0.57 |  | 0.80 |
| Prevalence |  |  |  |  |  |  |  |  |
| Chronic kidney disease (OR) |  | -0.29 (-0.64, -0.06) | 0.18 |  | 0.86 (-0.14, 1.86) | 0.09 |  | 0.38 |
| Microalbuminuria (OR) |  | -0.06 (-0.30, 0.18) | 0.12 |  | 0.27 (-0.10, 0.64) | 0.15 |  | 0.49 |

eGFR is the glomerular filtration rate estimated from serum creatinine by the CKD–EPI formula. Associations sizes were derived from mixed models that combined baseline and follow-up data (see Table 3). For chronic kidney disease (eGFR<60 mL/min/1.73 m2) and microalbuminuria (>3.5/>2.5 mg per mmol creatinine in women/men), association sizes are expressed as odds ratios (OR). Mixed models accounted for clustering of the baseline and follow-up data among participants living at the same address and were adjusted for sex, age (linear and squared term), mean arterial pressure, heart rate, body mass index, plasma glucose, total-to-HDL cholesterol ratio, γ‑glutamyltransferase, smoking, physical activity, socioeconomic class, and antihypertensive treatment (by drug class). An ellipsis indicates that the model did not converge. Association sizes, given with 95% confidence interval, were expressed for an interquartile range increment in the airborne particulate. Pint denotes the between-group significance of the difference in the association sizes.

**Supplementary Table 8.**

**Multivariable-adjusted cross-sectional associations of renal function with PM2.5 in various subgroups.**

| **Group analysed   Renal function trait** |  | **Estimate (95% CI)** | **P** |  | **Estimate (95% CI)** | **P** |  | **Pint** |
| --- | --- | --- | --- | --- | --- | --- | --- | --- |
| ***Sex*** |  | ***Women (N = 416)*** | |  | ***Men (N = 404)*** | |  |  |
| Serum creatinine (μmol/L) |  | 0.06 (-1.14, 1.26) | 0.92 |  | -0.03 (-1.48, 1.42) | 0.97 |  | 0.56 |
| eGFR (mL/min/1.73 m2) |  | 0.05 (-1.13, 1.23) | 0.93 |  | 0.28 (-0.88, 1.44) | 0.64 |  | 0.78 |
| Prevalence |  |  |  |  |  |  |  |  |
| Chronic kidney disease (OR) |  | -0.13 (-0.58, 0.32) | 0.56 |  | -0.23 (-0.76, 0.30) | 0.40 |  | 0.79 |
| Microalbuminuria (OR) |  | -0.07 (-0.32, 0.18) | 0.60 |  | 0.13 (-0.16, 0.42) | 0.39 |  | 0.83 |
| ***Age*** |  | ***<60 years (N = 588)*** | |  | ***≥60 years (N = 232)*** | |  |  |
| Serum creatinine (μmol/L) |  | 0.18 (-0.76, 0.76) | 0.71 |  | 0.25 (-2.13, 2.63) | 0.84 |  | 0.67 |
| eGFR (mL/min/1.73 m2) |  | 0.02 (-1.00, 1.00) | 0.97 |  | 0.03 (-1.50, 1.56) | 0.97 |  | 0.63 |
| Prevalence |  |  |  |  |  |  |  |  |
| Chronic kidney disease (OR) |  | -0.0004 (…) | … |  | -0.16 (-0.53, 0.21) | 0.41 |  | 0.85 |
| Microalbuminuria (OR) |  | -0.06 (-0.31, 0.18) | 0.61 |  | 0.27 (-0.16, 0.70) | 0.23 |  | 0.19 |
| ***Smoking status*** |  | ***Non-smokers (N = 685)*** | |  | ***Smokers (N = 135)*** | |  |  |
| Serum creatinine (μmol/L) |  | 0.38 (-0.76, 1.51) | 0.52 |  | -0.51 (-1.92, 0.90) | 0.49 |  | 0.47 |
| eGFR (mL/min/1.73 m2) |  | -0.27 (-1.27, 0.73) | 0.59 |  | 0.76 (-0.85, 2.37) | 0.37 |  | 0.60 |
| Prevalence |  |  |  |  |  |  |  |  |
| Chronic kidney disease (OR) |  | -0.12 (-0.45, 0.21) | 0.47 |  | -0.23 | NA |  | 0.57 |
| Microalbuminuria (OR) |  | 0.10 (-0.14, 0.34) | 0.39 |  | -0.31 (-0.60, -0.02) | 0.04 |  | 0.51 |
| ***Alcohol consumption*** |  | ***<5 g/day (N = 350)*** | |  | ***≥5 g/day (N = 470)*** | |  |  |
| Serum creatinine (μmol/L) |  | -0.03 (-1.36, 1.30) | 0.97 |  | 0.54 (-0.68, 1.76) | 0.38 |  | 0.77 |
| eGFR (mL/min/1.73 m2) |  | 0.09 (-1.05, 1.23) | 0.88 |  | -0.45 (-1.74, 0.84) | 0.49 |  | 0.81 |
| Prevalence |  |  |  |  |  |  |  |  |
| Chronic kidney disease (OR) |  | -0.30 (-0.65, 0.05) | 0.09 |  | 0.64 (-0.24, 1.52) | 0.15 |  | 0.35 |
| Microalbuminuria (OR) |  | -0.08 (-0.31, 0.15) | 0.49 |  | 0.23 (-0.14, 0.60) | 0.23 |  | 0.33 |

eGFR is the glomerular filtration rate estimated from serum creatinine by the CKD–EPI formula. Associations sizes were derived from mixed models that combined baseline and follow-up data (see Table 3). For chronic kidney disease (eGFR<60 mL/min/1.73 m2) and microalbuminuria (>3.5/>2.5 mg per mmol creatinine in women/men), association sizes are expressed as odds ratios (OR). Mixed models accounted for clustering of the baseline and follow-up data among participants living at the same address and were adjusted for sex, age (linear and squared term), mean arterial pressure, heart rate, body mass index, plasma glucose, total-to-HDL cholesterol ratio, γ‑glutamyltransferase, smoking, physical activity, socioeconomic class, and antihypertensive treatment (by drug class). Association sizes, given with 95% confidence interval, were expressed for an interquartile range increment in the airborne particulate. An ellipsis indicates that the model did not converge. Pint denotes the between-group significance of the difference in the association sizes.

**Supplementary Table 9.**

**Multivariable-adjusted associations between changes from baseline to follow-up in renal function and in the black carbon exposure.**

| **Group analysed   Renal function trait** |  | **N** | **Estimate (95% CI)** | **P** |  | **N** | **Estimate (95% CI)** | **P** |  | **Pint** |
| --- | --- | --- | --- | --- | --- | --- | --- | --- | --- | --- |
| ***Sex*** |  |  | ***Women (N = 328)*** | |  |  | ***Men (N = 325)*** | |  |  |
| Serum creatinine (%) |  | 328 | 0.38 (-0.85, 1.60) | 0.52 |  | 325 | -0.12 (-2.18, 1.93) | 0.90 |  | 0.43 |
| eGFR (%) |  | 328 | -0.42 (-2.18, 1.33) | 0.62 |  | 325 | -0.10 (-1.67, 1.46) | 0.89 |  | 0.58 |
| Incidence CKD (OR) |  | 24/293 | 0.72 (0.17, 1.28) | 0.01 |  | 24/307 | 0.30 (-0.34, 0.94) | 0.36 |  | 0.04 |
| ***Age*** |  |  | ***<60 years (N = 481)*** | |  |  |  | |  |  |
| Serum creatinine (%) |  | 481 | 0.15 (-1.40, 1.69) | 0.85 |  | 172 | -0.56 (-2.71, 1.59) | 0.60 |  | 0.24 |
| eGFR (%) |  | 481 | -0.53 (-1.80, 0.74) | 0.41 |  | 172 | 0.73 (-1.75, 3.21) | 0.55 |  | 0.20 |
| Incidence CKD (OR) |  | 8/470 | 0.07 (-0.59, 0.73) | 0.83 |  | 40/130 | 0.46 (-0.08, 1.01) | 0.09 |  | 0.69 |
| ***Smoking status*** |  |  | ***Non-smokers*** ***(N = 529)*** | |  |  |  | |  |  |
| Serum creatinine (%) |  | 529 | -0.10 (-1.03, 0.82) | 0.83 |  | 124 | 0.17 (-33.57, 33.92) | 0.96 |  | 0.24 |
| eGFR (%) |  | 529 | 0.16 (-1.04, 1.36) | 0.79 |  | 124 | -2.00 (-22.1, 18.14) | 0.43 |  | 0.17 |
| Incidence CKD (OR) |  | 41/482 | 0.31 (-0.14, 0.77) | 0.18 |  | 7/118 | 132.90 | NA |  | 0.16 |
| ***Alcohol consumption*** |  |  | ***<5 g/day (N = 380)*** | |  |  |  | |  |  |
| Serum creatinine (%) |  | 380 | 0.22 (-0.89, 1.34) | 0.69 |  | 273 | -0.11 (-2.47, 2.26) | 0.93 |  | 0.89 |
| eGFR (%) |  | 380 | -0.32 (-1.77, 1.14) | 0.67 |  | 273 | -0.14 (-2.04, 0.76) | 0.88 |  | 0.79 |
| Incidence CKD (OR) |  | 37/309 | 0.41 (-0.06, 0.87) | 0.08 |  | 11/291 | -0.12 | NA |  | 0.04 |

eGFR is the glomerular filtration rate estimated from serum creatinine by the CKD-EPI formula. For continuously distributed variables (serum creatinine and eGFR), changes were computed as the follow-up minus baseline values and expressed as a percentage of the baseline value. Incidence refers to new-onset chronic kidney disease (<60 mL/min/1.73 m2). N indicates the number of participants in the analysis or the number of new-onset cases/number of participants at risk. Associations accounted for clustering of data among participants living at the same address and were adjusted for sex, baseline age (linear and square term), socio-economic class, follow-up duration, baseline body mass index, the baseline value of and change during follow-up in mean arterial pressure, heart rate, plasma glucose, the total-to-HDL cholesterol ratio, γ‑glutamyltransferase, calories spent in physical activity, smoking status, and the intake of antihypertensive drugs (all drugs combined). Association sizes were expressed for an interquartile range increment in the airborne particulate. Pint denotes the between-group significance of the difference in the association sizes.

**Supplementary Table 10.**

**Multivariable-adjusted associations between changes from baseline to follow-up in renal function and in PM2.5 exposure.**

| **Group analysed   Renal function trait** |  | **N** | **Estimate (95% CI)** | **P** |  | **N** | **Estimate (95% CI)** | **P** |  | **Pint** |
| --- | --- | --- | --- | --- | --- | --- | --- | --- | --- | --- |
| ***Sex*** |  |  | ***Women (N = 416)*** | |  |  | ***Men (N = 404)*** | |  |  |
| Serum creatinine (%) |  | 328 | 0.45 (-0.81, 1.71) | 0.46 |  | 325 | -0.02 (-2.04, 1.99) | 0.98 |  | 0.72 |
| eGFR (%) |  | 328 | -0.51 (-2.32, 1.29) | 0.55 |  | 325 | -0.02 (-1.55, 1.50) | 0.98 |  | 0.43 |
| Incidence CKD (OR) |  | 24/293 | 0.56 (-0.07, 1.20) | 0.08 |  | 24/307 | 0.10 (-0.45, 0.65) | 0.72 |  | 0.17 |
| ***Age*** |  |  | ***<60 years (N = 481)*** | |  |  | ***≥ 60 years (N = 172)*** | |  |  |
| Serum creatinine (%) |  | 481 | 0.20 (-1.37, 1.77) | 0.80 |  | 172 | -0.17 (-2.26, 1.93) | 0.87 |  | 0.30 |
| eGFR (%) |  | 481 | -0.56 (-1.85, 0.73) | 0.39 |  | 172 | 0.30 (-2.12, 2.71) | 0.80 |  | 0.19 |
| Incidence CKD (OR) |  | 8/470 | -0.18 (-1.01, 0.66) | 0.67 |  | 40/130 | 0.27 (-0.25, 0.79) | 0.31 |  | 0.50 |
| ***Smoking status*** |  |  | ***Non-smokers*** ***(N = 529)*** | |  |  | ***Smokers (N = 124)*** | |  |  |
| Serum creatinine (%) |  | 529 | -0.10 (-1.03, 0.83) | 0.82 |  | 124 | 0.74 (-32.98, 34.46) | 0.83 |  | 0.30 |
| eGFR (%) |  | 529 | 0.23 (-0.97, 1.43) | 0.71 |  | 124 | -2.56 (-22.45, 17.33) | 0.35 |  | 0.19 |
| Incidence CKD (OR) |  | 41/482 | 0.14 (-0.31, 0.60) | 0.53 |  | 7/118 | 126.68 | NA |  | 0.54 |
| ***Alcohol consumption*** |  |  | ***<5 g/day (N = 380)*** | |  |  | ***≥5 g/day (N = 273)*** | |  |  |
| Serum creatinine (%) |  | 380 | 0.38 (-0.76, 1.52) | 0.51 |  | 273 | -0.09 (-2.39, 2.22) | 0.94 |  | 0.91 |
| eGFR (%) |  | 380 | -0.60 (-2.08, 0.88) | 0.42 |  | 273 | 0.09 (-1.76, 1.94) | 0.92 |  | 0.56 |
| Incidence CKD (OR) |  | 37/309 | 0.23 (-0.27, 0.72) | 0.36 |  | 11/291 | -0.26 | NA |  | 0.16 |

eGFR is the glomerular filtration rate estimated from serum creatinine by the CKD-EPI formula. For continuously distributed variables (serum creatinine and eGFR), changes were computed as the follow-up minus baseline values and expressed as a percentage of the baseline value. Incidence refers to new-onset chronic kidney disease (<60 mL/min/1.73 m2). N indicates the number of participants in the analysis or the number of new-onset cases/number of participants at risk. Associations accounted for clustering of data among participants living at the same address and were adjusted for sex, baseline age (linear and square term), socio-economic class, follow-up duration, baseline body mass index, the baseline value of and change during follow-up in mean arterial pressure, heart rate, plasma glucose, the total-to-HDL cholesterol ratio, γ‑glutamyltransferase, calories spent in physical activity, smoking status, and the intake of antihypertensive drugs (all drugs combined). Association sizes were expressed for an interquartile range increment in the airborne particulate. Pint denotes the between-group significance of the difference in the association sizes.

**Supplementary Table 11.**

**Multivariable-adjusted cross-sectional associations of renal function with black carbon and PM2.5 with reduced adjustment.**

| **Airborne particulate   Renal function** |  | **Baseline only  (n=820)** | |  | **Follow-up only  (n=653)** | |  | **Baseline and follow-up  (n=820)** | |
| --- | --- | --- | --- | --- | --- | --- | --- | --- | --- |
|  |  | **Estimate (95% CI)** | **P** |  | **Estimate (95% CI)** | **P** |  | **Estimate (95% CI)** | **P** |
| Black carbon |  |  |  |  |  |  |  |  |  |
| Serum creatinine (μmol/L) |  | 0.18 (‑0.77 to 1.14) | 0.70 |  | 0.07 (‑1.50 to 1.64) | 0.93 |  | 0.21 (‑0.89 to 1.31) | 0.71 |
| eGFR (mL/min/1.73 m2) |  | 0.03 (‑0.83 to 0.89) | 0.95 |  | ‑0.17 (‑1.27 to 0.93) | 0.77 |  | ‑0.01 (‑0.86 to 0.85) | 0.98 |
| Prevalence |  |  |  |  |  |  |  |  |  |
| eGFR <60 mL/min/1.73 m2 (%) |  | ‑0.03 (‑0.34 to 0.28) | 0.85 |  | 0.09 (‑0.24 to 0.42) | 0.60 |  | 0.05 (‑0.22 to 0.32) | 0.73 |
| Microalbuminuria (%) |  | 0.04 (-0.16, 0.24) | 0.70 |  | -0.00 (-0.20, 0.19) | 0.98 |  | 0.01 (-0.13, 0.15) | 0.91 |
| PM2.5 |  |  |  |  |  |  |  |  |  |
| Serum creatinine (μmol/L) |  | 0.14 (–0.82 to 1.10) | 0.78 |  | -0.07 (–1.64 to 1.50) | 0.93 |  | 0.12 (‑1.00, 1.24) | 0.84 |
| eGFR (mL/min/1.73 m2) |  | 0.04 (‑0.84 to 0.94) | 0.92 |  | ‑0.12 (‑1.22 to 0.98) | 0.83 |  | 0.01 (‑0.85 to 0.87) | 0.98 |
| Prevalence |  |  |  |  |  |  |  |  |  |
| eGFR <60 mL/min/1.73 m2 (%) |  | -0.01 (‑0.30 to 0.28) | 0.96 |  | -0.00 (‑0.32 to 0.31) | 0.99 |  | 0.01 (‑0.26) to 0.28 | 0.96 |
| Microalbuminuria (%) |  | 0.01 (-0.17, 0.19) | 0.89 |  | -0.01 (-0.19, 0.17) | 0.90 |  | -0.01 (-0.15, 0.13) | 0.91 |

eGFR is the glomerular filtration rate estimated from serum creatinine by the CKD–EPI formula. Associations sizes were derived from mixed models, which accounted for clustering of the baseline and follow-up data among participants living at the same address and were adjusted for sex, age (linear and squared term) and antihypertensive treatment (all drug classes combined). Association sizes, given with 95% confidence interval, were expressed for an interquartile range increment in the airborne particulate.

**Supplementary Table 12.**

**Multivariable-adjusted longitudinal associations of renal function with black carbon and PM2.5 with reduced adjustment.**

| **Group analysed**   **Renal function trait** | N |  | **Black carbon** | |  | **PM2.5** | |
| --- | --- | --- | --- | --- | --- | --- | --- |
|  |  |  | **Estimate (95% CI)** | **P** |  | **Estimate (95% CI)** | **P** |
| Serum creatinine (%) | 653 |  | ‑0.07 (‑1.68, 1.54) | 0.93 |  | ‑0.15 (‑1.76, 1.46) | 0.86 |
| eGFR (%) | 653 |  | ‑0.05 (‑1.17, 1.07) | 0.97 |  | ‑0.02 (‑1.14, 1.10) | 0.96 |
| Incidence of CKD (odds ratio) | 48/600 |  | 0.21 (-0.16, 0.59) | 0.27 |  | 0.07 (-0.29, 0.44) | 0.69 |

eGFR is the glomerular filtration rate estimated from serum creatinine by the CKD-EPI formula. For continuously distributed variables (serum creatinine and eGFR), changes were computed as the follow-up minus baseline values and expressed as a percentage of the baseline value. Incidence refers to new-onset chronic kidney disease (<60 mL/min/1.73 m2). N indicates the number of participants in the analysis or the number of new-onset cases/number of participants at risk. The change in the exposure to airborne particulate was expressed as the logarithmically transformed (log10) ratio of the follow-up-to-the-baseline value. Associations accounted for clustering of data among participants living at the same address and were adjusted for sex, baseline age (linear and square term), follow-up duration and the intake of antihypertensive drugs (all drug classes combined). Association sizes were expressed for an interquartile range increment in the airborne particulate.


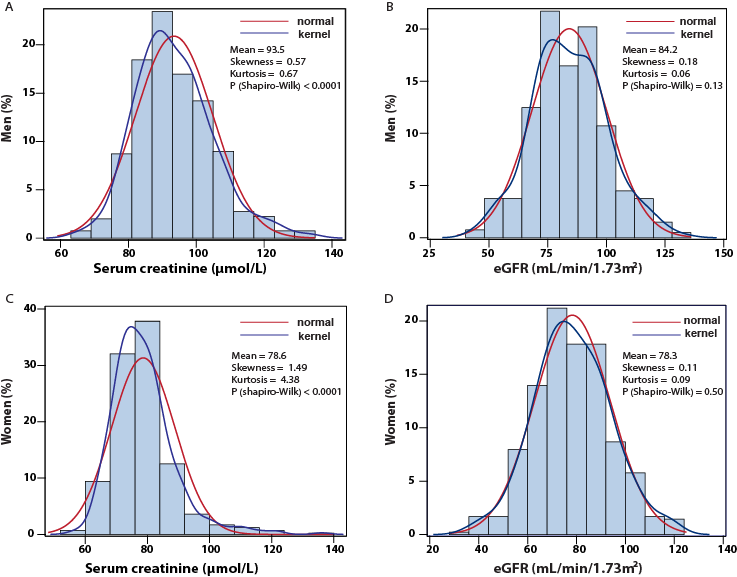


**Supplementary Figure S1.**

Distributions of serum creatinine (A, C) and the glomerular filtration rate estimated from serum creatinine (B, D) in 404 men (A, B) and in 416 women (C, D). The red and blue dotted lines represent the normal and kernel density distributions. The P values are for departure of the actually observed distribution from normality according to the Shapiro-Wilk statistic. Skewness and kurtosis were computed as the third and fourth moments about the mean divided by the cube of the standard deviation.


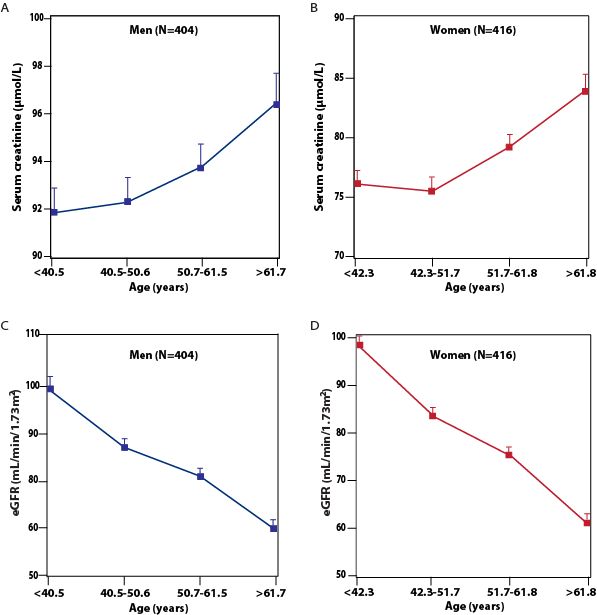


**Supplementary Figure** **S2.**

Association of serum creatinine (A, B) and the glomerular filtration rate estimated from serum creatinine (C, D) in 404 men and 416 women by fourths of the age distribution.
